# Comparative Genomic Analysis of Neutrophilic Iron(II) Oxidizer Genomes for Candidate Genes in Extracellular Electron Transfer

**DOI:** 10.3389/fmicb.2017.01584

**Published:** 2017-08-21

**Authors:** Shaomei He, Roman A. Barco, David Emerson, Eric E. Roden

**Affiliations:** ^1^Department of Geoscience, University of Wisconsin-Madison Madison, WI, United States; ^2^NASA Astrobiology Institute, University of Wisconsin Madison, WI, United States; ^3^Department of Bacteriology, University of Wisconsin-Madison Madison, WI, United States; ^4^Bigelow Laboratory for Ocean Sciences East Boothbay Harbor, ME, United States; ^5^Department of Earth Sciences, University of Southern California Los Angeles, CA, United States

**Keywords:** neutrophilic Fe(II) oxidation, extracellular electron transfer (EET), porin-cytochrome *c* complex (PCC), cytochrome *c*, multicopper oxidase, genomics

## Abstract

Extracellular electron transfer (EET) is recognized as a key biochemical process in circumneutral pH Fe(II)-oxidizing bacteria (FeOB). In this study, we searched for candidate EET genes in 73 neutrophilic FeOB genomes, among which 43 genomes are complete or close-to-complete and the rest have estimated genome completeness ranging from 5 to 91%. These neutrophilic FeOB span members of the microaerophilic, anaerobic phototrophic, and anaerobic nitrate-reducing FeOB groups. We found that many microaerophilic and several anaerobic FeOB possess homologs of Cyc2, an outer membrane cytochrome *c* originally identified in *Acidithiobacillus ferrooxidans*. The “porin-cytochrome *c* complex” (PCC) gene clusters homologous to MtoAB/PioAB are present in eight FeOB, accounting for 19% of complete and close-to-complete genomes examined, whereas PCC genes homologous to OmbB-OmaB-OmcB in *Geobacter sulfurreducens* are absent. Further, we discovered gene clusters that may potentially encode two novel PCC types. First, a cluster (tentatively named “PCC3”) encodes a porin, an extracellular and a periplasmic cytochrome *c* with remarkably large numbers of heme-binding motifs. Second, a cluster (tentatively named “PCC4”) encodes a porin and three periplasmic multiheme cytochromes *c*. A conserved inner membrane protein (IMP) encoded in PCC3 and PCC4 gene clusters might be responsible for translocating electrons across the inner membrane. Other bacteria possessing PCC3 and PCC4 are mostly Proteobacteria isolated from environments with a potential niche for Fe(II) oxidation. In addition to cytochrome *c*, multicopper oxidase (MCO) genes potentially involved in Fe(II) oxidation were also identified. Notably, candidate EET genes were not found in some FeOB, especially the anaerobic ones, probably suggesting EET genes or Fe(II) oxidation mechanisms are different from the searched models. Overall, based on current EET models, the search extends our understanding of bacterial EET and provides candidate genes for future research.

## Introduction

Microbes play key roles in the redox cycling of iron (Fe), the fourth most abundant element as well as the most abundant redox active metal in the Earth's crust. Microorganisms capable of oxidizing ferrous iron [Fe(II)] were recognized as early as the nineteenth century (Winogradsky, 1888); however, a deeper understanding of the biological process of Fe(II) oxidation and the diversity of mechanisms in Fe(II)-oxidizing bacteria (FeOB) have yet to be attained. Over the past few decades, the cosmopolitan distribution of FeOB in nature has been demonstrated in numerous Fe(II)-rich environments, including freshwater, marine and subsurface habitats (Emerson et al., 2010; Hedrich et al., 2011; Dubinina and Sorokina, 2014). This accumulated evidence highlights the potential global impact of these microbes in the biogeochemistry of Fe.

Phylogenetically, most FeOB are affiliated with Proteobacteria. A few other phyla, such as Chlorobi, Nitrospirae, Firmicutes and Actinobacteria, also harbor FeOB. Being physiologically diverse, members of FeOB include lithoautotrophs, heterotrophs and mixotrophs. For our purposes, we define lithoautotrophic FeOB as those organisms that rely on Fe(II) as a primary energy source to drive CO_2_-fixation; heterotrophic FeOB are those bacteria that oxidize Fe(II) but do not derive an energetic benefit; while mixotrophic FeOB may gain energy from the oxidation, but may utilize organic matter to make biomass. In addition to these general metabolic categories, there are a number of different physiologies related to adaptations to pH, electron acceptor, and energy source, thus FeOB can be classified into acidophilic aerobes, neutrophilic microaerobes, neutrophilic anaerobic phototrophs, and neutrophilic anaerobic nitrate-reducers (Hedrich et al., 2011).

Due to the insolubility of the reaction product, ferric iron [Fe(III)], at neutral pH, neutrophilic FeOB require electron transport systems that are strategically positioned to avoid intracellular Fe precipitation. Therefore, the oxidation of aqueous Fe(II) most likely occurs at the cell outer surface through extracellular electron transfer (EET). Alternatively, this reaction can potentially occur in the periplasm with reaction products being pumped to the outside (Schädler et al., 2009). EET is also required when the electron-donating Fe(II) is in the solid-phase.

EET systems have been extensively studied in model Fe(III)-reducing bacteria (FeRB), such as *Geobacter sulfurreducens* and *Shewanella oneidensis*, for which electrons from the oxidation of organic substrates are passed to extracellular Fe(III) minerals. One strategy of EET in Fe(III) reduction is via electron shuttles, which are redox-active molecules (such as flavins/riboflavins and humic acids) either secreted by the microbes or naturally present in the environment that act to shuttle electrons between membrane-associated redox proteins and extracellular Fe-oxides (Lovley et al., 1998; Okamoto et al., 2014). Therefore, a direct physical contact between the Fe mineral substrate and redox enzyme is not required when using electron shuttles. Another EET strategy is through direct contact. For example, Type IVa pili from *G. sulfurreducens* (Reguera et al., 2005) and outer membrane and periplasmic extensions from *S. oneidensis* (Gorby et al., 2006; Pirbadian et al., 2014) can form highly conductive biological “nanowires” for long-distance electron transfer. Alternatively, EET could be achieved through a direct contact between extracellular Fe and a dedicated redox protein at the cell outer surface. Such redox-active enzymes are often cytochromes *c* (Cyt *c*) (Richter et al., 2012; Shi et al., 2012, 2016; White et al., 2016), and less often, multicopper oxidases (MCO) (Mehta et al., 2006).

**Figure 1:**
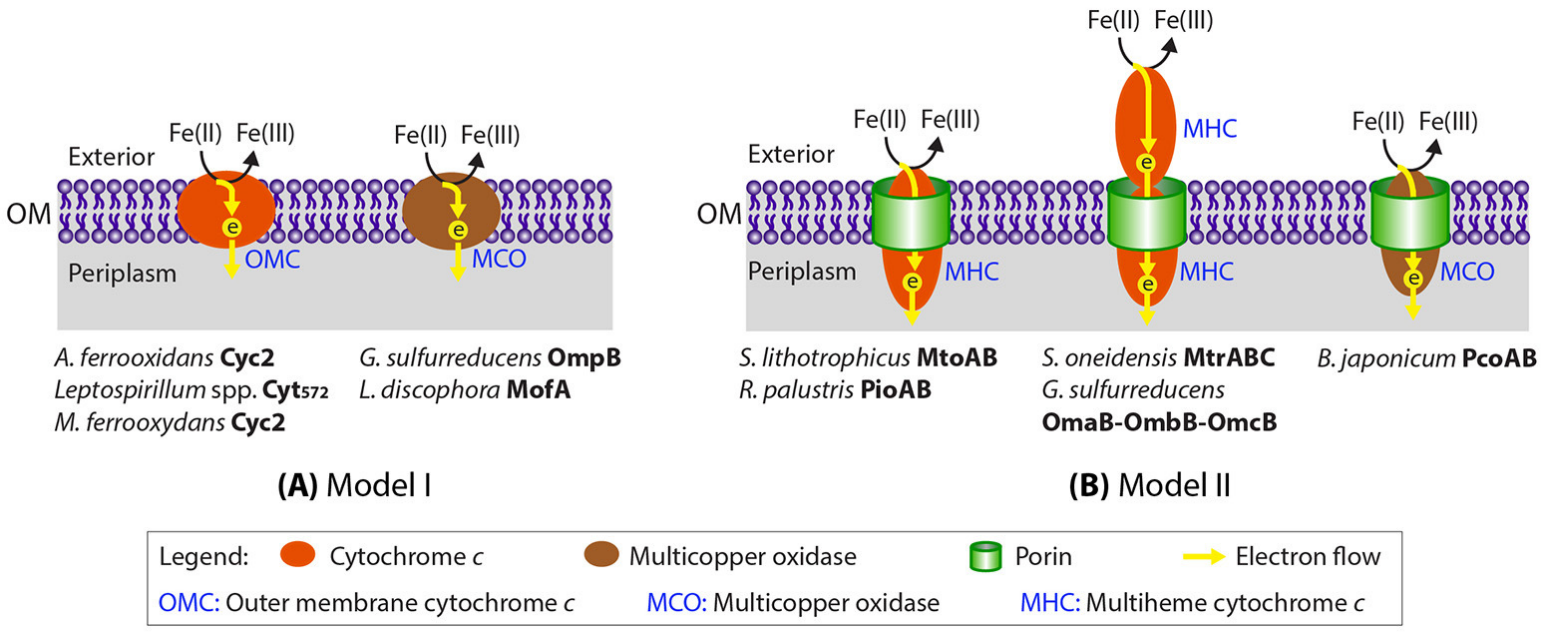
Two generalized models proposed for Fe(II) oxidation at the cell outer surface through a dedicated Fe(II) oxidase (Cyt *c* or multicopper oxidase). Previously proposed EET proteins in Fe(II) oxidation/reduction and Mn(II) oxidation that fall into the two general models are indicated. Notably, the EET system involving PcoAB proteins was proposed for *B. japonicum* solely based on bioinformatic analysis without any experimental evidence.

Like the Fe(III)-reducing *Geobacter* and *Shewanella* spp., most neutrophilic FeOB are Gram-negative bacteria with the inner and outer membranes separated by a periplasmic space, thus EET systems also need to span the periplasm to bridge the outer membrane to the inner membrane. Based on current knowledge of EET in Fe redox reactions, we condensed the different Gram-negative bacterial Fe redox systems that involve a direct contact between extracellular Fe and a dedicated outer membrane Fe oxidoreductase into two generalized models (Figure 1). In one model, the Fe oxidase (and reductase) is secreted as an outer-membrane/extracellular protein (Figure 1A). Examples include Cyc2, an outer membrane Cyt *c* which was originally identified in the acidiphilic FeOB *Acidithiobacillus ferrooxidans* (Castelle et al., 2008) with distant homologs later identified in neutrophilic microaerobic *Mariprofundus* spp. (Barco et al., 2015), outer membrane multiheme Cyt *c*, such as OmcE, OmcS, and OmcZ in *G. sulfurreducens* (Leang et al., 2003; Mehta et al., 2005), and outer membrane MCO, such as OmpB and OmpC in *G. sulfurreducens* (Mehta et al., 2006; Holmes et al., 2008). In the other model, the Fe oxidase (and reductase), which is often a multiheme cytochrome *c* (MHC), is secreted into the periplasm, and embedded into a porin on the outer membrane to directly or indirectly interact with extracellular Fe (Figure 1B). This latter system was referred to as the “porin-cytochrome *c* protein complex” (PCC) (Richardson et al., 2012). Examples of PCC include PioA (a decaheme Cyt *c*) and PioB (a porin) in the phototrophic FeOB *Rhodopseudomonas palustris* TIE-1 (Jiao and Newman, 2007) and their homologous MtoAB proteins in microaerophilic FeOB *Sideroxydans lithotrophicus* ES-1 (Liu et al., 2012; Shi et al., 2012; Emerson et al., 2013). Sometimes, an extracellular MHC is also a component of the PCC system, such as the MtrABC complex in *S. oneidensis* (Beliaev and Saffarini, 1998) and the later discovered PCC complex in *G. sulfurreducens* that consists of a porin-like protein (OmbB/OmbC), a periplasmic octaheme Cyt *c* (OmaB/OmaC), and an outer-membrane dodecaheme Cyt *c* (OmcB/OmcC) (Liu et al., 2014). More recently, a porin and an associated periplasmic MCO in some FeOB were hypothesized to be candidate EET proteins that form a complex analogous to porin-periplasmic Cyt *c* (He et al., 2016).

Facilitated by the availability of FeOB genomes, a significant progress has been made in searching for candidate EET systems in neutrophilic FeOB (Liu et al., 2012; Emerson et al., 2013; Barco et al., 2015; Kato et al., 2015). As part of these efforts, we sequenced the genomes of five FeOB strains isolated from the Hanford subsurface (Benzine et al., 2013) and one FeOB strain associated with marine basalt oxidation (Swanner et al., 2011). We also previously recovered draft genomes of putative FeOB from a pyrite-oxidizing enrichment culture (Percak-Dennett et al., 2017) and nitrate-dependent Fe(II)-oxidizing (NDFO) enrichment culture KS described by Straub et al. (1996) through metagenomic analyses (He et al., 2016). These, together with the increasing number of publicly available FeOB genomes, provide an opportunity to systematically search for genes that might be involved in EET during Fe(II) oxidation at circumneutral pH through comparative genomic analysis.

In this study we searched more than 70 neutrophilic FeOB genomes, including isolate genomes, single-cell amplified genomes, and metagenome-assembled genomes (MAGs), spanning neutrophilic members of the microaerophilic, anaerobic nitrate-reducing, and phototrophic FeOB that range from obligate lithoautotrophs to mixotrophs/heterotrophs. We focused on EET mechanisms that involve a direct contact between redox-active proteins and extracellular Fe, i.e., dedicated outer membrane Fe(II) oxidases or conductive pili/nanowires. For the former case, we searched for the two generalized Fe(II) oxidase models summarized in Figure 1: (1) outer-membrane/extracellular Cyt *c* or MCO, and (2) porin-periplasmic Cyt *c* complex (i.e., PCC) or porin-periplasmic MCO complex. Our analysis synthesizes information on EET genetic systems across the largest number of neutrophilic FeOB taxa to date, with the goal of discovering novel putative EET gene clusters, extending our understanding of bacterial EET, and providing a list of interesting candidate genes for future omics research.

## Materials and methods

### Genomes studied

We previously recovered draft genomes of putative FeOB from metagenomes constructed from a pyrite-oxidizing enrichment culture (Percak-Dennett et al., 2017) and two nitrate-dependent Fe(II)-oxidizing (NDFO) enrichment cultures (Straub et al., 1996; He et al., 2016). We also sequenced genomes of five FeOB strains isolated from the Hanford subsurface (Benzine et al., 2013) through the Joint Genome Institute's Microbial Isolates sequencing program (project 1016754) and one FeOB strain associated with marine basalt oxidation (Swanner et al., 2011). With other publicly available neutrophilic FeOB genomes, a total of 73 genomes were included, and all are available at Integrated Microbial Genomes (IMG, https://img.jgi.doe.gov/cgi-bin/m/main.cgi). The IMG genome taxon ID, physiology, taxonomy and references of these FeOB are listed in Supplementary Table 1.

### Phylogenetic tree construction for FeOB genomes

We constructed a phylogenetic tree of all included FeOB genomes with concatenated alignments of conserved phylogenetic markers using the PhyloPhlAn analysis pipeline (Segata et al., 2013). All protein sequences translated *in silico* from these genomes were input to PhyloPhlAn for extracting and individually aligning the conserved phylogenetic marker proteins. The alignments were then concatenated by PhyloPhlAn. The concatenated alignment was used to construct a maximum likelihood phylogenetic tree using PhyML 3.0 (Guindon et al., 2010), with the LG substitution model (Le and Gascuel, 2008) and the gamma distribution parameter estimated by PhyML.

### Bioinformatic analysis

For cytochromes *c*, heme-binding sites were counted by the canonical CXXCH motif, as well as the CX_3_CH and CX_4_CH motifs. Protein cellular location was predicted using CELLO v.2.5 (http://cello.life.nctu.edu.tw) (Yu et al., 2006) and PSORTb v.3.0 (http://www.psort.org/psortb) (Yu et al., 2010). The beta-barrel structure of outer membrane proteins was predicted by PRED-TMBB (http://bioinformatics.biol.uoa.gr//PRED-TMBB) (Bagos et al., 2004). Sequence repeats within a protein were predicted by RADAR (http://www.ebi.ac.uk/Tools/pfa/radar/) (Heger and Holm, 2000).

### Search for EET genes

To search for known EET gene homologs, BLASTP and/or HMMsearch for known protein families were conducted. To search for novel putative EET genes, we first identified all Cyt *c* and MCO genes in the genome, and then bioinformatically predicted their cellular locations to see if they are outer membrane/extracellular proteins. To search for gene clusters that potentially encode porin-Cyt *c* or porin-MCO complex, we first identified genes that encode periplasmic Cyt *c* or periplasmic MCO, and then checked the five genes upstream and five genes downstream of the focal Cyt *c* or MCO gene to see if any of the downstream or upstream genes encode an outer membrane beta-barrel protein that likely forms a porin. In all the known “porin-cytochrome *c* complex” (PCC) gene clusters, the porin-coding gene is next to a Cyt *c* gene; therefore checking five genes up- and down-stream of the Cyt *c* or MCO gene should be exploratory enough to capture candidate gene clusters analogous to the known ones. Candidate genes and gene clusters were further evaluated to see if they are likely involved in EET.

### Phylogenetic tree construction for individual proteins

Phylogenetic trees were constructed for Cyc2-like proteins, porin proteins in PCC3 and porin proteins in PCC4. Protein sequences were aligned with MUSCLE (Edgar, 2004) and trimmed to exclude columns which contain gaps for more than 30% of the included sequences. Maximum likelihood phylogenetic trees were constructed using PhyML 3.0 (Guindon et al., 2010), with the LG substitution model (Le and Gascuel, 2008) and the gamma distribution parameter estimated by PhyML. Bootstrap values were calculated based on 100 replicates or otherwise indicated. Trees were visualized with Dendroscope (v3.2.10) with midpoint root.

## Results and discussion

### Overview

**Figure 2:**
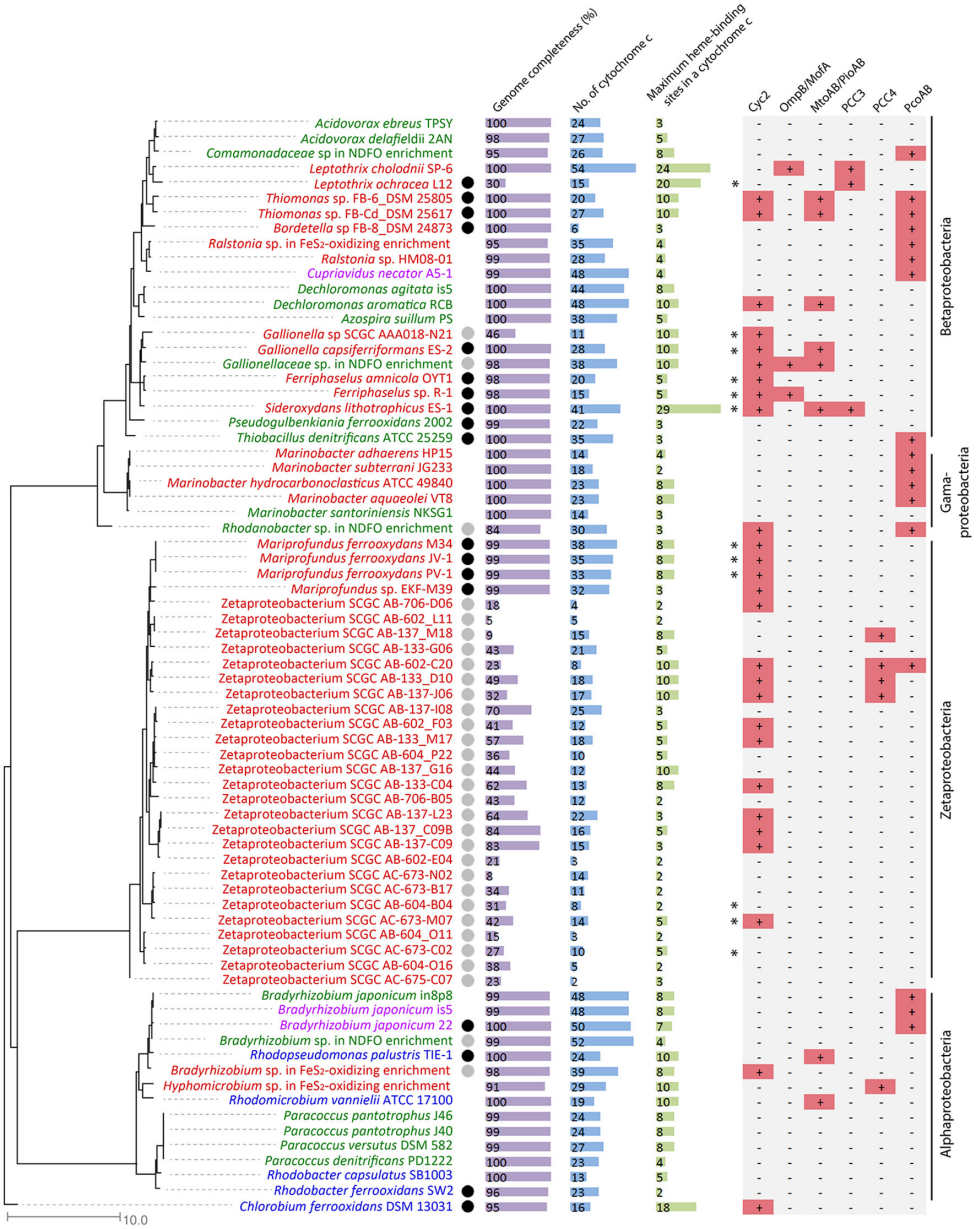
Phylogenetic tree constructed from FeOB genomes, with genome completeness, total number of Cyt *c* genes in the genome, the maximal number of heme-binding sites in a Cyt *c*, and putative EET genes indicated. Genomes labeled in red, green, and blue are microaerobic, anaerobic nitrate-dependent, and anaerobic phototrophic FeOB, respectively. Genomes labeled in purple are FeOB that can use both oxygen and nitrate as electron acceptors for Fe(II) oxidation. Experimentally verified obligate lithoautotrophic FeOB are labeled with a black circle, and FeOB that are likely capable of lithoautotrophic Fe(II) oxidation based on genome information and their closely related FeOB isolated from similar environments are labeled with a gray circle. Genomes containing alternative complex III (ACIII) are labeled with ^*^ on the left of the Cyc2 column.

We searched 73 genomes, including the Alpha-, Beta-, Gamma- and Zeta-proteobacteria and a Chlorobia species, spanning neutrophilic FeOB from the microaerophilic, anaerobic phototrophic, and anaerobic nitrate-reducing groups, and ranging from obligate lithoautotrophs to mixotrophs/heterotrophs (Figure 2, Supplementary Table 1). Some of them are single-amplified genomes (SAGs) of putative FeOB retrieved from Fe(II)-oxidizing environments or MAGs recovered from metagenomes of Fe(II)-oxidizing enrichment cultures. Notably, 43 genomes are complete or close-to-complete (estimated completeness >95%), while the remaining 30 genomes (including two MAGs and 28 SAGs, 26 of which were Zetaproteobacteria SAGs) have estimated genome completeness ranging from 5 to 91%. Therefore, the lack of an EET genetic system in these incomplete genomes should be interpreted with caution, as the absence of a gene could be due to the incomplete genome coverage. Despite this limitation, these incomplete genomes also provided valuable insights when putative EET systems were identified or when a pattern of their EET gene distribution was observed.

Cyt *c* and especially MHC are important components of the well-studied EET pathways involved in Fe redox reactions and their genes are highly represented in model FeRB genomes. For example, genomes of *G. sulfurreducens* and *S. oneidensis* are markedly rich in Cyt *c* genes (e.g., 111 in *G. sulfurreducens* and 42 in *S. oneidensis*) and many of these are MHCs (Methé et al., 2003; Meyer et al., 2004). MHCs, particularly those with large numbers of hemes, may be able to form molecular “wires” for conducting electrons from the periplasmic space across the outer membrane (Clarke et al., 2011; Bewley et al., 2013). We therefore counted the Cyt *c* genes and their heme-binding motifs in FeOB genomes. The total number of Cyt *c* genes in the 43 complete and close-to-complete FeOB genomes ranges from 6 to 54 (Figure 2), with a median number of 27. Therefore, although some FeOB genomes also contain large numbers of Cyt *c* genes, being Cyt *c*-rich is not a common feature of neutrophilic FeOB genomes. The maximal number of heme-binding motifs among all MHCs within a FeOB genome ranges from 2 to 29 (Figure 2). Notably, only 10 out of the 43 complete and close-to-complete genomes contain MHCs with at least 10 heme-binding sites. This suggests that either many neutrophilic FeOB do not employ MHC-based systems (similar to *A. ferrooxidans*, which does not possess Cyt *c* with more than two hemes), or if they do, there is likely to be only a limited number of types used for Fe(II) oxidation. This further suggests that other, diverse EET systems may be involved. Based on our current understanding of EET pathways involved in Fe redox reactions, we identified some putative EET systems, which are summarized in Figure 2, with their occurrences in each genome indicated, and these systems are discussed in detail below.

### Outer membrane cytochrome *c*, Cyc2

In the acidiphilic FeOB *A. ferrooxidans*, the outer membrane Cyc2 was suggested to shuttle electrons from Fe(II) to periplasmic rusticyanin, which relays electrons to two periplasmic cytochromes *c*, CycA1 and Cyc1, respectively; the former then transfers electrons to the inner membrane *bc*_1_ complex for NADH production, and the latter transfers electrons to the inner membrane terminal oxidase for energy generation (Castelle et al., 2008). Recently, a distant homolog of Cyc2, named Cyc2_*PV*−1_, was identified in the neutrophilic microaerobic FeOB *Mariprofundus ferrooxydans* PV-1 as a highly expressed outer membrane protein, along with the periplasmic Cyc1_*PV*−1_ and either the inner membrane alternative complex III (ACIII) or the *bc*_1_complex (Barco et al., 2015). Based on these findings, a model involving Cyc2_*PV*−1_, Cyc1_*PV*−1_ and ACIII was proposed as an EET conduit for *M. ferrooxydans*. Further comparative genomic analysis revealed the presence of Cyc2 and ACIII in all neutrophilic microaerophilic FeOB with complete genomes available, including *S. lithotrophicus* ES-1, *Gallionella capsiferriformans* ES-2 and *Ferriphaselus* spp. within the *Gallionellaceae* family, and *Mariprofundus* spp. (Kato et al., 2015), along with some other related marine Zetaproteobacterial FeOB SAGs (Field et al., 2015).

We previously identified Cyc2 homologs in draft genomes of a *Gallionellaceae* sp. and a *Rhodanobacter* sp. recovered from the nitrate-dependent Fe(II)-oxidizing (NDFO) enrichment culture KS (He et al., 2016), as well as in a *Bradyrhizobium* sp. from a pyrite-oxidizing enrichment culture (Percak-Dennett et al., 2017). In the latter case, *Bradyrhizobium* Cyc2 gene is clustered with genes encoding a high-potential iron-sulfur protein and three periplasmic cytochromes *c*, including homologs of Cyc1 and CycA1, and these proteins likely work together with Cyc2 to relay electrons from the outer membrane to the inner membrane (Percak-Dennett et al., 2017).

**Figure 3:**
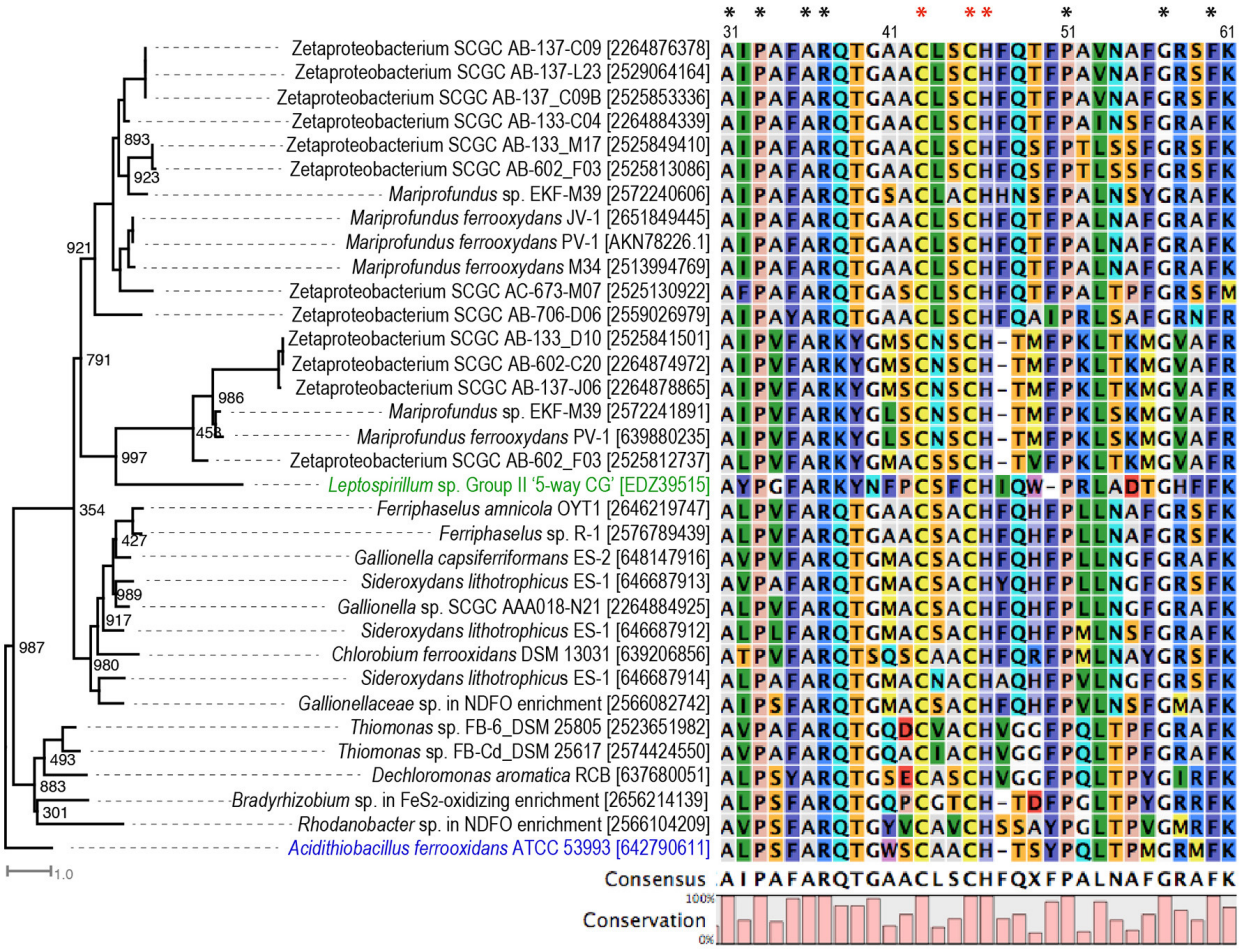
Phylogenetic tree of Cyc2 in *A. ferrooxidans* (labeled in blue), Cyt_572_ in *Leptospirillum* sp. (labeled in green), and their homologs in the studied FeOB genomes, with their conserved N-terminal sequences aligned. Numbers in the bracket are IMG gene ID or GenBank accession numbers. The sequence numbering is according to the amino acid position in *A. ferrooxidans* ATCC 53993. Amino acids conserved in all sequences in the tree are indicated with ^*^, and the conserved residues in the heme-binding motif are labeled with red ^*^. Bootstrap values were calculated based on 1,000 replicates.

In our expanded search of other neutrophilic FeOB genomes, we found Cyc2-like genes in two *Thiomonas* strains that are capable of microaerophilic Fe(II) oxidation under slightly acidic conditions (pH 5.5) (Fabisch et al., 2011), in the anaerobic nitrate-reducing *Dechloromonas aromatica* RCB, and the phototrophic *Chlorobium ferrooxidans* (Figures 2, 3). Cyc2 homologs were also identified in two newly isolated obligate chemolithoautotrophic FeOB, including Zetaproteobacteria strain TAG-1 and *Mariprofundus* sp. DIS-1 (Mumford et al., 2016). Notably, although not found in the phototrophic FeOB *R. palustris* TIE-1, Cyc2 homologs are present in several other *R. palustris* strains not known for Fe(II) oxidation (Figure 3). In addition, Cyc2-like genes are also present in endosymbionts of the deep-sea tubeworms *Riftia pachyptila, Tevnia jerichonana* (Barco et al., 2015; Kato et al., 2015), and *Ridgeia piscesae*. These endosymbionts might be able to conduct EET as we identified another putative EET conduit (i.e., PCC3) in their genomes, as discussed later.

Overall, Cyc2 is widely present in known obligate lithotrophic FeOB, although not universally present in all FeOB (Figure 2). In particular, Cyc2 is conserved in the obligate chemolithoautotrophic FeOB within freshwater *Gallionellaceae* spp. and marine Zetaproteobacteria related to *Mariprofundus* spp., forming two distinct clusters in the phylogenetic tree (Figure 3). The presence of Cyc2 homologs and the ability to oxidize Fe(II) suggests a common Fe(II)-oxidizing ancestor for these chemolithoautotrophic microbes. It also suggests close past interactions, perhaps in estuaries, between two classes of Proteobacteria that currently seem to inhabit different environmental niches: freshwater and marine habitats. By contrast, the presence of Cyc2 among chemolithoautotrophic *Bradyrhizobium* FeOB genomes is not consistent (Figure 2), and the Fe(II) oxidation capability is not conserved within the broader *Bradyrhizobium* genus, suggesting that Cyc2, in this case, might be acquired via a more recent horizontal gene transfer. Interestingly, many bacteria not known for Fe(II) oxidation also possess Cyc2 homologous genes (Supplementary Table 2). It is possible that these bacteria may be able to oxidize Fe(II), or Cyc2 may be utilized by these bacteria in EET processes other than Fe(II) oxidation, and these possibilities need to be further tested.

It is worth noting that Cyc2-like proteins in neutrophilic FeOB are only distant homologs of Cyc2 in *A. ferrooxidans*. It was previously reported that Cyc2 in *A. ferrooxidans* only shares significant homologies at the N-terminus with Cyc2_PV−1_ (Barco et al., 2015) and Cyt_572_, an outer membrane Fe(II) oxidase in the acidiphilic FeOB *Leptospirillum* spp. (Jeans et al., 2008). The alignment of Cyc2-like proteins from neutrophilic FeOB along with Cyc2 and Cyt_572_ also indicates their homology at the N-terminus, with a number of conserved amino acids, including a conserved heme-binding motif (Figure 3). Despite the lack of significant sequence homology in the C-terminal regions of these proteins, they were consistently predicted to be a beta-barrel structure likely forming a porin domain for Cyc2, Cyt_572_, and Cyc2_PV−1_ (Yarzábal et al., 2002; Jeans et al., 2008; Barco et al., 2015). Therefore, White et al. (2016) referred to Cyc2 as a fused porin-cytochrome protein with the N-terminal cytochrome domain forming a “plug” within the beta-barrel structure.

In addition to Cyc2, ACIII was suggested as another shared feature of microaerobic FeOB (Kato et al., 2015). However, in the expanded list of microaerobic FeOB genomes that contain Cyc2, a few of them do not possess ACIII, such as *Mariprofundus* sp. EKF-M39 and *Bradyrhizobium* sp. in the pyrite-oxidizing enrichment (Figure 2). In addition, ACIII is not identified in anaerobic nitrate-dependent or phototrophic Cyc2-containing FeOB genomes (Figure 2). Therefore, the conservation of ACIII is restricted to microaerobic freshwater *Gallionellaceae* and marine *M. ferrooxydans* strains. If Cyc2-like proteins are involved in EET in the ACIII-lacking FeOB, a different inner membrane enzyme (complex), such as the canonical Complex III (*bc*_1_complex) may be used to perform the role, as has been found in *A*. *ferrooxidans* (Bruscella et al., 2007).

Genes encoding extracellular/outer membrane Cyt *c* other than Cyc2-like proteins are also present in some of the FeOB genomes, and these extracellular/outer membrane Cyt *c* are often MHCs and may be components of known or novel porin-MHC complexes, as discussed later.

### Extracellular/outer membrane MCO (OmpB/MofA)

**Figure 4:**
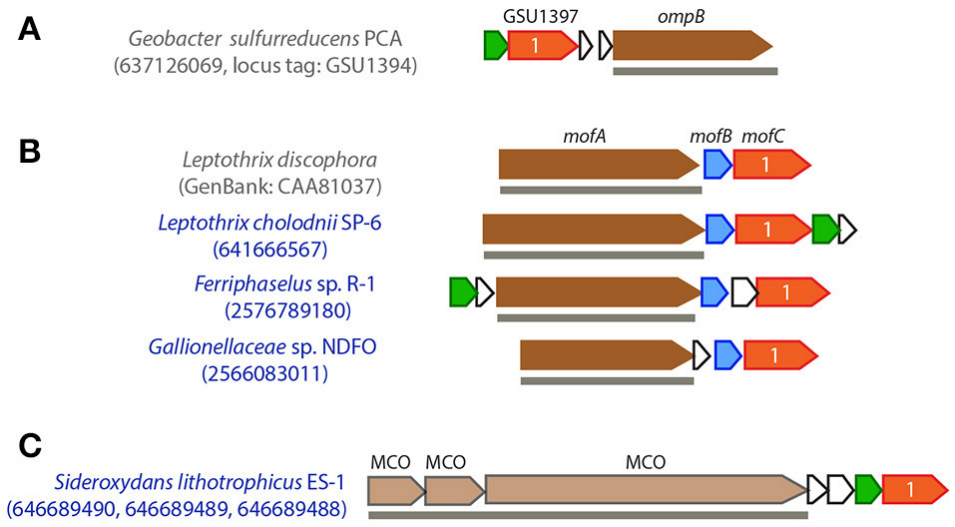
Gene clusters that contain OmpB/MofA homologs in *G. sulfurreducens* **(A)** and metal oxidizers **(B)**, or other extracellular/outer membrane multicopper oxidase (MCO) encoding genes **(C)**. FeOB names are labeled in blue. IMG gene IDs (or GenBank accession number) for the MCO genes are shown in the parenthesis for locating these gene clusters. OmpB/MofA homologs are colored in dark brown and other extracellular/outer membrane MCO genes are colored in light brown. Genes in red are homologous to *mofC* and contain a Cyt *c* domain, with the number of heme-binding sites indicated; genes in blue are *mofB* homologs; genes in green encode putative copper chaperones belonging to an electron transport protein SCO1-SenC family. Horizontal lines below genes indicate predicted outer membrane/extracellular protein coding genes.

In *G. sulfurreducens*, an outer membrane-associated MCO, namely OmpB (locus tag GSU1394, Figure 4A), was shown to be required for the reduction of poorly soluble Fe(III) oxides (Mehta et al., 2006; Qian et al., 2007). As a unique MCO, OmpB contains Fe(III)-binding motifs (EXXE) and a fibronectin Type III domain, which often plays a role in bacterial adhesion, and thus is possibly involved in attaching to Fe(III) (oxyhydr)oxides (Mehta et al., 2006). OmpB is homologous to a putative manganese oxidase (MofA) in the heterotrophic Mn(II) oxidizer *Leptothrix discophora* (Corstjens et al., 1997), where *mofA* forms an operon with *mofB* and *mofC* (Figure 4B). By sequence analysis, it was hypothesized that MofB, a peptidylprolyl isomerase functioning as a protein-folding chaperone, was involved in the folding and activation of MofA, and MofC, a putative Cyt *c*, may function in concert with MofA and MofB to transfer electrons during Mn(II) oxidation (Brouwers et al., 2000), although their functions have yet to be experimentally elucidated. The *ompB*-containing gene cluster in *G. sulfurreducens* also has a gene encoding a distant homolog of MofC (GSU1397, with protein sequence 29% identical to MofC), but lacks a *mofB* homolog; instead it contains a putative copper chaperone gene (GSU1398, belonging to the SCO1/SenC family) next to the *mofC* homologous gene (Figure 4A).

Genes encoding OmpB/MofA homologs were identified in *Gallionellaceae* sp. from the NDFO enrichment culture KS (He et al., 2016), as well as in the heterotrophic FeOB *L. cholodnii* SP-6 and autotrophic FeOB *Ferriphaselus* sp. R-1 (Figure 2). In these FeOB, the OmpB/MofA homologs are predicted to be secreted extracellularly, and the *ompB*/*mofA*-like gene is clustered with a *mofB*- and a *mofC*-like gene, and in two cases, is also clustered with a putative copper chaperone gene (Figure 4B). Likewise, these MofC-like proteins also contain a Cyt *c* domain and a periplasmic binding domain. As the functions of OmpB and MofA are yet to be further studied and verified, it is less clear whether their homologous gene clusters in FeOB might have a similar role in metal redox reactions analogous to OmpB and MofABC hypothesized for *G. sulfurreducens* and *L. discophora* respectively. Nevertheless, we tentatively propose them as candidate EET genes for future research.

In addition to MofA/OmpB homologs, three tandem MCO-coding genes, along with a *mofC*-like and a copper chaperon gene in the same cluster, were found in *S. lithotrophicus* ES-1 (Figure 4C). Similar to OmpB/MofA, the three MCO proteins are predicted to be located extracellularly. However, as they are not homologous to previously suggested metal oxidoreductases, fewer clues are available for their functional inference.

### MtrAB/MtoAB/PioAB-type PCC

In the other general model involving a dedicated Fe oxidoreductase, instead of being an extracellular or outer membrane protein, the oxidoreductase is secreted to the periplasm, and embedded into a porin within the outer membrane to directly or indirectly interact with extracellular Fe. Such a “porin-cytochrome *c* protein complex” (PCC) model was first identified in the FeRB *S. oneidensis* (Beliaev and Saffarini, 1998). In this case, an inner membrane tetraheme Cyt *c* (CymA) transfers electrons to MtrA, where “mtr” stands for metal reduction. MtrA is a decaheme Cyt *c* embedded in MtrB, a porin with 28 transmembrane regions that forms a sheath for MtrA in the outer membrane. MtrA then shuttles electrons to the outer membrane decaheme Cyt *c* (MtrC), which further transfers electrons to extracellular Fe minerals (Richardson et al., 2012; Shi et al., 2012). Homologs of MtrAB complex were also identified in several FeOB, including PioAB in *R. palustris* TIE-1, where “pio” stands for photosynthetic Fe(II) oxidation (Jiao and Newman, 2007) and MtoAB in *S. lithotrophicus* ES-1 and *G. capsiferriformans* ES-2, where “mto” stands for metal oxidation (Liu et al., 2012; Shi et al., 2012; Emerson et al., 2013) (Figure 1B).

**Figure 5:**
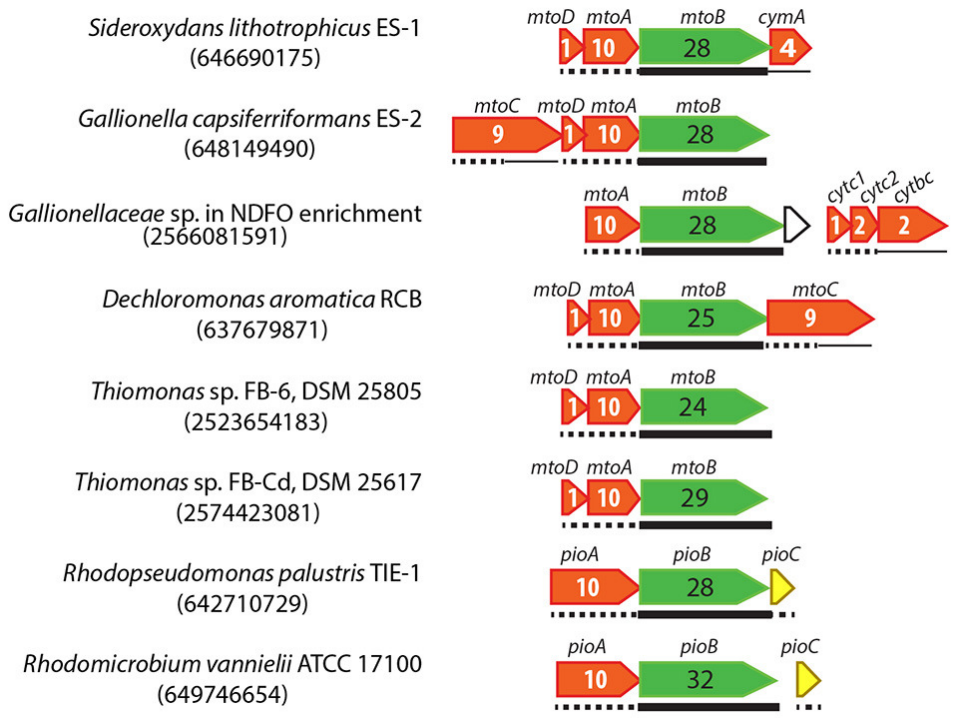
Gene clusters containing MtoAB or PioAB homologs in FeOB genomes. IMG gene IDs for the porin-coding genes are shown in the parenthesis for locating these gene clusters. Porin-coding genes are colored in green, with the number of transmembrane motifs indicated in the gene. Cyt *c* genes are colored in red, with the number of heme-binding sites indicated. Predicted cellular locations of encoded proteins are shown by different line types under the genes, with thin lines, dashed lines, and thick lines indicating inner membrane, periplasm, and outer membrane, respectively.

Previously we found homologs of MtoAB in *Gallionellaceae* sp. from the NDFO enrichment culture KS (He et al., 2016). MtoAB/PioAB homologs are also present in several other FeOB, including MtoAB in anaerobic nitrate-reducing *D. aromatica* RCB and microaerobic *Thiomonas* sp. strains FB-6 and FB-Cd, and PioAB in phototrophic *Rhodomicrobium vannielii* ATCC 17100 (Figure 2). In all cases, the core protein, MtoA/PioA, is a periplasmic Cyt *c* with 10 heme-biding sites, and the other core protein, MtoB/PioB, is an outer membrane beta-barrel with 24–32 transmembrane regions, likely forming a porin (Figure 5).

For strains ES-1 and ES-2, Shi et al. (2012) suggested that a periplasmic monoheme Cyt *c* (MtoD, which is encoded in the same *mto* operon) relays electrons from MtoA to an inner membrane Cyt *c* (tetraheme CymA in ES-1 and nonaheme MtoC in ES-2 encoded in the *mto* operon) (Figure 5). *Gallionellaceae* sp. in the NDFO enrichment culture KS does not contain MtoD, although it is a close relative of ES-1 and ES-2. Instead, downstream of its *mto* operon is a three-gene operon consisting of two small periplasmic Cyt *c-*coding genes (referred to as Cyt*c*1 and Cyt*c*2 respectively) and a gene encoding an inner membrane protein (IMP) fused by a cytochrome *b*561 domain at the N-terminus and a diheme Cyt *c* at the C-terminus (tentatively referred to as Cyt*bc*) (Figure 5). We previously hypothesized that, in this NDFO *Gallionellaceae* sp., the two small periplasmic Cyt *c* (Cyt*c*1 and Cyt*c*2 respectively) may act similarly to MtoD in ES-1 and ES-2, and relay electrons from MtoA to the inner membrane Cyt*bc*, which is predicted to be a quinone-active enzyme similar to CymA in ES-1 and MtoC in ES-2 (He et al., 2016). The *mto* operon in *D. aromatica* RCB is similar to that in ES-2 and consists of *mtoABCD*, therefore likely using the same mechanism as in ES-2. The *mto* operon in the two *Thiomonas* strains only contains *mtoABD*, and lacks any obvious inner membrane redox gene upstream or downstream.

Instead of using a Cyt *c* to relay electrons in the periplasm, the phototrophic *R. palustris* TIE-1 transfers electrons from PioA to PioC, a high-potential iron-sulfur protein, which then donates electrons to the inner membrane phototrophic reaction center for photosynthetic energy generation (Bird et al., 2014). Similarly, a complete *pioABC* operon is also present in phototrophic *R. vannielii* (Figure 5), probably functioning in the same way as in *R. palustris* TIE-1.

Besides the homologous MtrAB/PioAB/MtoAB system, a phylogenetically unrelated PCC was later identified in *G. sulfurreducens* as an EET conduit in Fe(III) reduction (Liu et al., 2014). Despite the lack of any sequence homology, this PCC also consists of a porin-like protein, a periplasmic octaheme Cyt *c*, and an outer-membrane dodecaheme Cyt *c*, encoded by *ombB, omaB*, and *omcB* respectively (or by their downstream homologous *ombC-omaC-omcC* gene cluster that likely arose from gene duplication) (Liu et al., 2014). Further, this PCC genetic system was identified in a number of bacteria that have the ability to perform redox reactions involving insoluble substrates or products, which would also require EET (Shi et al., 2014). In our current search, homologs of the OmbB-OmaB-OmcB complex are not present in any of the FeOB included in this analysis. However, we found gene clusters that may potentially encode two novel PCC systems discussed below.

### Putative PCC3, a novel PCC system?

**Figure 6:**
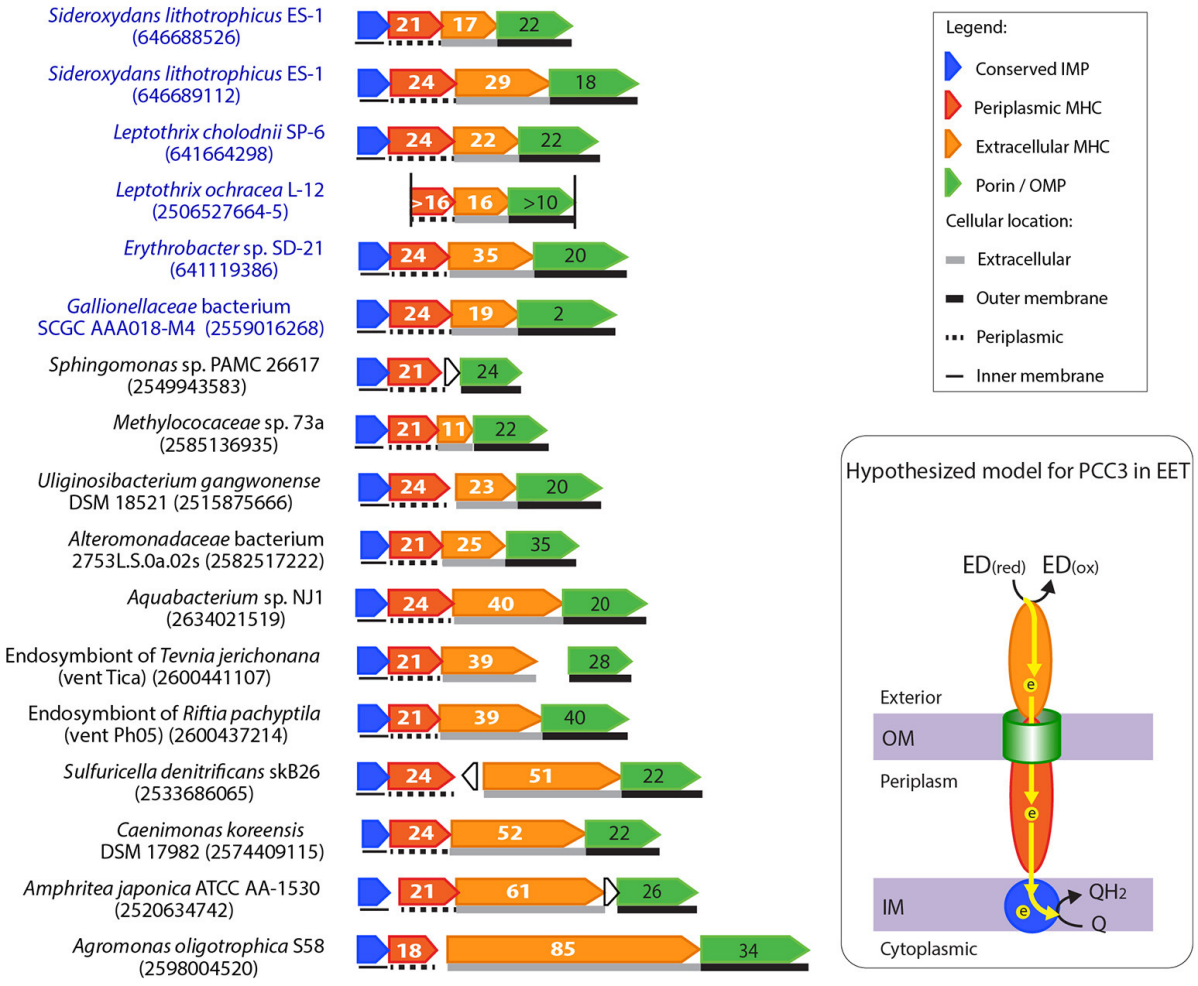
Gene clusters potentially encoding a novel porin-cytochrome *c* complex, “PCC3.” The names of bacteria known for metal oxidation are labeled in blue, with IMG gene IDs for the porin-coding gene indicated in the parenthesis. Porin-coding genes (green) have the number of transmembrane motifs indicated, and extracellular and periplasmic MHC genes (orange and red respectively) have the number of heme-binding sites indicated. The two vertical lines in the *Leptothrix ochracea* L-12 gene cluster indicate the two ends of the contig, and its porin-coding gene was wrongly split to two (2506527664-5) due to a sequencing error (homopolymer of A's), which caused a frame shift and a stop codon in the middle. A model for the electron flow between the extracellular donor to the inner membrane quinone pool by PCC3 was hypothesized, with proteins colored according to the colors of their encoding genes on the left. ED_(red)_ and ED_(ox)_ stand for the reduced and oxidized forms of the electron donor.

A common feature of the PCC gene clusters is the co-occurrence of a porin-coding and a periplasmic MHC-coding gene. Besides these two essential components, genes encoding extracellular MHC, other periplasmic or inner membrane redox proteins may be present in the same gene cluster. Based on this general genetic organization, we searched for putative PCC proteins that do not share homology with the two established PCC systems (i.e., MtrAB/MtoAB/PioAB and OmbB-OmaB-OmcB). We found a gene cluster that may encode such a novel PCC in *S. lithotrophicus* ES-1, *Leptothrix cholodnii* SP-6, and *L. ochracea* L12 (Figure 2), and tentatively referred to it as “PCC3” to distinguish it from the two known PCC systems. The PCC3 gene cluster consists of an outer membrane protein with a predicted beta-barrel structure likely forming a porin, an extracellular MHC, a periplasmic MHC, and a conserved IMP without an apparent functional annotation (Figure 6).

As no specific function has been proposed for these gene clusters, we searched all available bacterial genomes in IMG for their homologs, and found their presence in a number of Alpha-, Beta-, and Gamma-proteobacteria. Figure 6 shows some examples of these PCC3 gene clusters (For the complete list of PCC3-containing bacteria, see Supplementary Tables 3, 4, which also list their physiology/habitat information, and features of their component genes, respectively). Among these bacteria, a *Gallionellaceae* sp. and *Erythrobacter* sp. are Fe(II) and Mn(II) oxidizers respectively, while the rest are not known for metal redox reactions. Despite the substantial difference in gene lengths and numbers of heme-binding sites in their MHCs, these clusters have a largely conserved organization of genes that encode porin, extracellular MHC, periplasmic MHC, and IMP.

Notably, most porins in PCC3 have a large number (e.g., up to 42) of transmembrane regions (Figure 6, Supplementary Table 4), likely forming a large pore size (see examples of 2D-representation of the predicted beta-barrel structure of the porin in Supplementary Figure 1). The porin phylogenetic tree indicated a grouping pattern largely based on their hosting bacterial taxonomy and habitats (Supplementary Figure 2). Most of the Alphaproteobacteria containing this complex are plant symbionts from paddy soil or symbionts of rock-dwelling lichens; the Gammaproteobacteria include animal symbionts or free-living bacteria from marine environments, such as hydrothermal vents and marine sediments, while many of the Betaproteobacteria are of freshwater origin (Supplementary Table 3). Some of these habitats are often rich in Fe (e.g., hydrothermal vents, paddy soils, and marine or freshwater sediments), providing a potential niche for EET to occur. Interestingly, the PCC3-containing gammaproteobacterial autotrophic endosymbionts of deep-sea tubeworms *R. pachyptila, T. jerichonana*, and *R. piscesae* in hydrothermal vents also have Cyc2-like genes as discussed earlier. Therefore, these symbionts might be able to conduct redox reactions involving EET. This is intriguing in light of the finding of Zetaproteobacteria epibionts of the hydrothermal vent shrimp *Rimicaris exoculata* that were suggested to oxidize soluble Fe(II) to Fe(III) oxyhydroxides in order to prevent the Fe(II) toxicity to the host (Jan et al., 2014). Other PCC3-containing bacteria that also possess Cyc2-like genes include *Uliginosibacterium gangwonense* DSM 18521, *Bradyrhizobium* sp. STM 3843, *Dechloromonas* sp. JJ, *Methylotenera versatilis* 301, *Methylococaceae* sp. 73a, *Sulfuricella denitrificans* skB26, and *Aquabacterium* sp. NJ1.

MHCs in PCC3 usually have remarkably large numbers of heme-binding sites, with the median number of heme-binding sites of 34 and 21 for the extracellular MHC and periplasmic MHC, respectively (Figure 6, Supplementary Table 4). For example, *Amphritea atlantica* DSM 18887 isolated from a hydrothermal vent has extracellular MHC and periplasmic MHC with 63 and 21 heme-binding sites respectively. Another extremely large number of heme-binding sites was found in the plant symbiont *Agromonas oligotrophica* S58, in which the extracellular MHC has 85 heme-binding sites. To our knowledge this is the largest number of heme-binding sites reported in a single protein. A previous systematic investigation of MHCs in a total of 594 completely sequenced prokaryotic genomes in 2010 (Sharma et al., 2010) reported a maximum of 45 heme-binding sites in a single protein. Six of the MHCs reported in our study exceeded that number (Supplementary Table 4). The large numbers of hemes in these MHC proteins suggest their primary role is to conduct electrons, as supported by the HemeBIND (Liu and Hu, 2011) prediction that most of their amino acids are involved in binding and coordinating hemes. This is also reflected by the exceptionally low amino acid to heme ratios in these proteins, ranging from 22 to 39, with the median of 26 (Supplementary Table 4). For comparison, a systematic survey of prokaryote MHCs indicated that the typical amino acid to heme ratio for MHCs is ~60–70 ± 30, with the smallest ratio (~33) observed in the decaheme MtrA homologs (Sharma et al., 2010). As MtrA in *S. oneidensis* is structurally shaped like an elongated wire with an estimated maximal dimension of ~100 Å, spanning a substantial portion of the periplasmic space (Firer-Sherwood et al., 2011), the low amino acid to heme ratio was suggested to be a feature of electronically conductive MHC wires (Bewley et al., 2013). Therefore, the low ratios in the PCC3 MHCs reported here are consistent with a similar function as conductive “molecular wires”. Compared to MtrA, their longer protein lengths and larger numbers of heme-binding sites may suggest that they are capable of spanning the dimension between the two membranes of Gram-negative bacteria.

Another interesting feature of the MHCs in PCC3 is the low complexities of their primary sequences. First, these MHCs are highly enriched in several amino acids. For example, the extracellular MHCs are rich in lower molecular-weight amino acids with minimal side chains, such as threonine, serine, alanine, and glycine, in addition to cysteine and histidine, which are within the heme-binding CXXCH motif. The small side chains of these amino acids may allow the proteins to coil with less spatial restriction and avoid the shielding of heme-binding sites due to steric hindrance. Second, these MHC sequences contain large numbers of repeat regions, with each repeat unit containing at least one heme-binding site. For example, Supplementary Figure 1 highlighted the sequence repeats and heme-binding sites of the PCC3 MHCs in *L. cholodnii* SP-6 and an endosymbiont of *R. pachyptila* from a hydrothermal vent, respectively. These features are consistent with the hypothesized formation of a wire structure tightly packed with a large number of stacked hemes electronically connected for rapid electron transfer.

The conserved IMP component in PCC3 is about 300 amino acids long, and was annotated as a hypothetical protein with no assigned function. We hypothesize that the IMP in PCC3 is likely a redox protein to relay electrons across the inner membrane (see more discussion in the “Inner Membrane Proteins in PCC3 and PCC4” Section). Therefore, the PCC3 gene cluster has hallmarks consistent with the formation of an EET conduit with electronically connected components spanning the entire Gram-negative cell wall.

Although it seems likely that PCC3 gene clusters are involved in EET based on the interesting features of their component proteins and the habitats of their hosting bacteria, it is not clear whether they are specifically involved in Fe(II) oxidation or may participate in other EET-required redox reactions, as PCC3 is only present in a few of the known FeOB genomes. Nevertheless, the discovery of these novel MHCs with extremely large numbers of heme-binding sites may suggest an unexplored EET pathway widely distributed in nature beyond Fe(II) oxidation.

### Putative PCC4, another novel PCC system?

**Figure 7:**
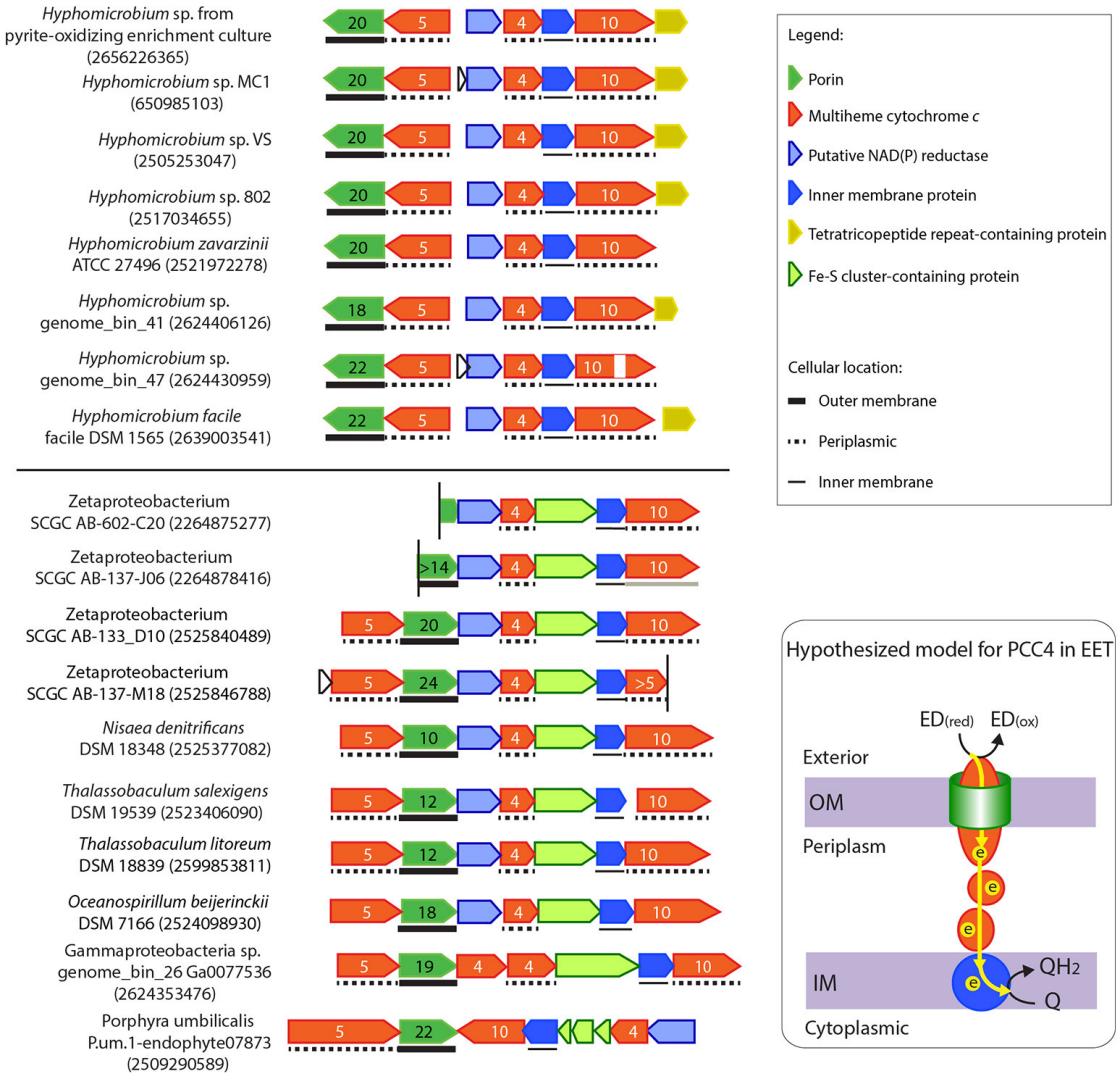
Gene clusters potentially encoding another novel porin-cytochrome *c* complex, “PCC4.” IMG gene IDs for the porin-coding genes are shown in the parenthesis for locating these gene clusters. Porin-coding genes (green) have numbers of transmembrane motifs indicated, and MHC genes (red) have numbers of heme-binding sites indicated. The vertical line indicates the end of a contig. In *Oceanospirillum beijerinckii* DSM 7166, the Fe-S cluster containing gene and the IMP gene were annotated as one gene in IMG, but could have been split into two as shown here. A horizontal line between the two gene clusters indicates the boundary of Subgroups I and II. A model for the electron flow between the extracellular donor to the inner membrane quinone pool by PCC4 was hypothesized, with proteins colored according to the colors of their encoding genes on the left. ED_(red)_ and ED_(ox)_ stand for the reduced and oxidized forms of the electron donor.

Another type of putative PCC gene cluster (tentatively referred to as “PCC4”) was found in the *Hyphomicrobium* sp. draft genome retrieved from a pyrite-oxidizing enrichment culture and in four marine Zetaproteobacteria FeOB genomes (Figure 2). PCC4 includes genes encoding a porin, three periplasmic MHCs with 4, 5, and 10 heme-binding sites respectively, a conserved IMP, and other redox-active proteins. A sequence similarity search in genomes available at IMG expanded the list of bacteria that contain the PCC4 gene cluster (Figure 7). Interestingly, four draft genomes recovered from a drinking water filter were among the list, including a Gammaproteobacteria sp. and three *Rhizobiales* spp. that are phylogenetically closest to *Hyphomicrobium* spp. (Figure 7, Supplementary Figure 3). *Hyphomicrobium* spp. were suggested to play a role in Mn(II) oxidation in freshwater sediments (Stein et al., 2001; Palermo and Dittrich, 2016). In water treatment, filters are often used to remove Fe(II) and Mn(II) by their biological oxidation to form precipitates of Fe(III) and Mn(IV) oxyhydroxides [see reviews on this topic (Gounot, 1994; Emerson and De Vet, 2015)]. Therefore, it is intriguing to discover the PCC4 gene cluster in *Hyphomicrobium* spp. and bacteria retrieved from the drinking water filter.

Unlike PCC3, no extracellular MHC is present in PCC4, indicating that this gene cluster may be more analogous to the MtoAB in ES-1 (Liu et al., 2012) and PioAB in *R. palustris* TIE-1 (Jiao and Newman, 2007) that do not have extracellular MHC components. As in PCC3, the conserved IMPs in PCC4 are also likely involved in electron transfer across the inner membrane (see more discussion in the “Inner Membrane Proteins in PCC3 and PCC4” Section), and thus also likely form an EET conduit with the porin and periplasmic MHCs.

Based on the gene organization and content, these PCC4 gene clusters can be classified into Subgroups I and II. The two subgroups are based on having different translation directions of the component genes, and Subgroup I lacks the Fe-S cluster (pfam13247)-containing gene, which is upstream of the IMP gene in Subgroup II. In addition, while the redox genes upstream of the tetraheme Cyt *c* comprise the same component domains, they have different domain organization between the two subgroups (see domain architectures in Supplementary Figure 4). The grouping pattern based on gene organization and content is also largely reflected in the phylogenetic tree of the porin sequences (Supplementary Figure 3). Subgroup I bacteria exclusively consists of *Hyphomicrobium* spp., and are mostly from freshwater environments, such as water and wastewater treatment systems, saturated soil, and lakes (Supplementary Table 5). Subgroup II contains Zeta-, Gamma- and other Alpha-proteobacteria, and Planctomycetes, mostly from marine environments, including hydrothermal vents and coastal seawater (Supplementary Table 5). These habitats are consistent with where FeOB have been found.

### Inner membrane proteins in PCC3 and PCC4

**Figure 8:**
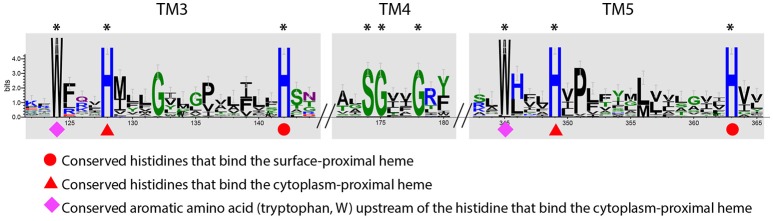
Sequence conservation logo generated from the alignment of a total of 47 IMP sequences from PCC3 and PCC4. The logo was generated using the WebLogo tool (v3.4) (Crooks et al., 2004), with the stack height indicating the degree of conservation. Conserved amino acid residues on the 3rd, 4th, and 5th transmembrane (TM) regions (TM3, TM4, and TM5, respectively) are labeled with ^*^. The logo is not continuous, as only the conserved regions are shown. The symbols under the conserved amino acids indicate the functions of that residue according to Zhang et al. (2013).

IMPs between PCC3 and PCC4 do not share significant overall sequence homology, indicating that they belong to different protein families. However, when analyzing the predicted secondary structure and conserved residues, we were intrigued to find some interesting similarities between the two IMP groups. All IMPs in PCC3 and PCC4 were predicted to have five transmembrane helices, and all have a conserved **SG**XX**G** motif on the fourth transmembrane helix and two conserved **W**X_3_**H**X_13_**H** motifs on the third and fifth helix respectively (Figure 8), probably suggesting important functions of these motifs and a similar role of the IMP in these two genetic systems, due to their conservation in both IMP groups.

Notably, the feature of two **H**X_13_**H** motifs located on the third and fifth TM helix respectively is also an attribute of the ferric reductase transmembrane component domain (FRD, pfam01794), which is often found in ferric reductase (FRE) and NAD(P)H oxidase (NOX), and is structurally similar to the cyt*b* subunit of the cytochrome *bc*_1_ complex (Complex III) (Zhang et al., 2013). In the FRD domain or the cyt*b* subunit, the four histidine residues in the two conserved **H**X_13_**H** motifs are involved in the binding of two *b*-type hemes. Therefore, the IMPs in PCC3 and PCC4 are also likely redox proteins with two *b*-type hemes, and possibly act as a quinone-active cyt*b* similar to the cyt*b* subunit of the *bc*_1_ complex or the cyt*b* subunit of the [NiFe]-hydrogenase I complex, which are able to exchange electrons with the quinone pool in the inner membrane (Vignais et al., 2001; Gross et al., 2004). Based on this, we hypothesized models for PCC3 and PCC4 in EET, with the IMP component facilitating electron transfer across the inner membrane (Figures 6, 7).

### Other multiheme cytochrome *c*

In addition to the MHCs in MtoAB/PioAB, PCC3, and PCC4, we also investigated the functional annotation of MHCs with more than three heme-binding sites in FeOB genomes due to the general importance of MHCs in EET. Most of these MHCs have predicted functions including the tetraheme cytochrome *c* subunit of nitrate or TMAO reductase, the pentaheme cytochrome *c* in ACIII, as well as the octaheme cytochrome *c* in tetrathionate reductases and hydroxylamine oxidoreductases, etc. However, a small number of predicted periplasmic MHCs do not have apparent functional assignment, and immediately downstream of these MHC genes is often a hypothetical protein, which is predicted to be an IMP (Supplementary Table 6). Despite the difference in the number of heme-binding sites and the sequence length of MHCs, their associated IMPs are conserved. Although most of these IMPs are shorter than, and do not share a significant sequence homology with IMPs in PCC3 and PCC4, they also have five transmembrane helices and two **H**X_13_**H** motifs on the third and fifth helix, and thus may be functionally related to IMPs in PCC3 and PCC4. Similar to PCC4, in most cases, there is another redox protein in the same gene cluster (Supplementary Table 6). However, different from PCC3 and PCC4, there is no porin-coding gene in the same gene cluster, except for *L. cholodnii* SP-6 (porin gene IMG ID 641666910, with 24 transmembrane regions) and *C. ferrooxidans* DSM 13031 (porin gene IMG ID 639205727, with 25 transmembrane regions). Therefore, most of these MHCs cannot be exposed on the outer membrane, and it is less likely that they are involved in EET.

### Porin-periplasmic MCO?

An interesting question is whether a porin-MCO system functionally and structurally analogous to the porin-Cyt *c* complex exists in these FeOB genomes, as MCO is another type of enzyme involved in metal redox reactions, such as Fe(III) reduction (Mehta et al., 2006), Mn(II) oxidation (Soldatova et al., 2012), and Cu(I) oxidation, which confers copper resistance (Chaturvedi and Henderson, 2014). Previously, a gene encoding a predicted periplasmic MCO was found in the same operon as a porin-like gene in Fe(II)-oxidizing *Bradyrhizobium japonicum* strain 22 isolated from Hanford, and based on bioinformatic analysis these two genes were hypothesized to form a porin-MCO complex for EET (L. Shi and E. Roden, unpublished data). Indeed, such a putative porin-MCO gene cluster is present in two other Fe(II)-oxidizing *B. japonicum* strains (is5 and in8p8) isolated from Hanford, whereas it was absent in another 20 *B. japonicum* strains not known for Fe(II) oxidation (Supplementary Table 7). The homologous porin-MCO gene cluster is also present in a *Comamonadaceae* sp. and a *Rhodanobacter* sp. from the NDFO enrichment culture KS (He et al., 2016), and was identified in a number of FeOB, including microaerobic and nitrate-reducing FeOB that span the Alpha-, Beta-, Gamma- and Zeta-proteobacteria (Figure 2, Supplementary Figure 5).

The predicted periplasmic MCO and its associated porin-like protein are not novel, but belong to the PcoA and PcoB protein families, respectively. *PcoAB* were initially identified as component genes of a plasmid-borne copper-resistance operon (*pco*) in *Escherichia coli* (Lee et al., 1990), and are homologous to *CopAB* in a *Pseudomonas syringae* copper-resistance operon (*cop*) (Cha and Cooksey, 1991). Among component genes in the *pco* (or *cop*) operon, *pcoAB* (or *copAB*) are essential to the resistance (Mellano and Cooksey, 1988), and therefore are more conserved than other genes in the operon (Rensing and Grass, 2003).

It was suggested that PcoA detoxifies copper by oxidizing siderophores, which subsequently chelate and sequester Cu(I), or by directly oxidizing the extremely toxic Cu(I) to less toxic Cu(II) (Rensing and Grass, 2003; Chaturvedi and Henderson, 2014), and PcoB may interact with PcoA to export Cu(II) across the outer membrane (Chaturvedi and Henderson, 2014). PcoA may have a broad substrate spectrum. For example, PcoA in *P. aeruginosa* was able to oxidize Fe(II) to Fe(III) for subsequent transport using Fe(III) transporters for Fe acquisition (Huston et al., 2002). Therefore, it is plausible that PcoA in these FeOB can also oxidize Fe(II), while PcoB, which is predicted to form an outer membrane beta-barrel structure usually with 10–12 transmembrane motifs (Supplementary Figure 5), might allow PcoA to access to the outer surface for EET to occur, or facilitate the export of oxidized Fe in the periplasm across the outer membrane.

Taken together, we hypothesize that, in addition to the previously identified role in copper resistance, PcoAB might form a porin-MCO system, in which PcoA oxidizes Fe(II). However, it is not clear whether such a putative system is for metal efflux or acts as a part of the EET conduit coupled with energy generation.

### Conductive nanowires

Alternative to the direct contact between extracellular Fe and a dedicated outer membrane Fe(II) oxidase, EET might be achieved through conductive nanowires analogous to those known for *Geobacter* and *Shewanella*, that allow for long-range EET over micrometer distances (Reguera et al., 2005; Gorby et al., 2006). Nanowires in *Shewanella* are tubular structures formed by outer membrane and periplasmic extensions with decaheme cytochromes MtrC and OmcA localized along the membrane to conduct electrons (Pirbadian et al., 2014). However, neither MtrC nor OmcA homologs were identified in the FeOB genomes included in our study. By contrast, nanowires in *Geobacter* are pilin-based (Reguera et al., 2005). These conductive pili are members of the Type IVa family of pili, comprising of a structural protein unit, PilA. PilA proteins from *Geobacter* spp. have conserved N-terminal α-helix sequences of Type IVa pilin, but their C-terminal regions are truncated, lacking a C-terminal globular head that is present in PilA from non-conductive pili. As a result, the *Geobacter* PilA proteins are considerably shorter than typical PilA (Reardon and Mueller, 2013). For example, the mature PilA from *G. sulfurreducens* (GSU1496) is only 61 amino acids long after the cleavage by the prepilin peptidase, much shorter than mature PilA from non-conductive *Neisseria gonorrhoeae* (158 amino acids) and *Pseudomonas aeruginosa* (144 amino acids). The lack of the large C-terminal globular head was suggested to promote electronic coupling and charge transport (Feliciano et al., 2012). Further, five aromatic residues (F24, Y27, Y32, F51, and Y57) on the C-terminal region of the mature PilA of *G. sulfurreducens* are critical to confer the metallic-like conductivity by their pi-pi orbital stacking (Vargas et al., 2013; Feliciano et al., 2015). These unique structural configurations of its pilin proteins account for the ability of *Geobacter* PilA to transfer electrons.

Genes encoding PilA are present in about 40% of the studied FeOB genomes. However, except for a PilA in Zetaproteobacterium SCGC AB-706-B05, the predicted lengths of these PilA are more than 100 amino acids when mature, much longer than the PilA from *G. sulfurreducens*. Aligning these PilA sequences with PilA structures from *G. sulfurreducens* (PDB 2M7G), *N. gonorrhoeae* (PDB 2HIL) and *P. aeruginosa* (PDB 1OQW) indicates that these PilA have multiple beta strands in the C-terminus, therefore, likely forming a C-terminal globular head as the typical PilA instead of a conductive one. The exception, a PilA from Zetaproteobacterium SCGC AB-706-B05 (Gene locus tag K258DRAFT2_00603, IMG ID 2559026090) has a mature length of 52 amino acids. However, it lacks three of the five aromatic residues required for the conductivity. Therefore, based on these analyses, it is unlikely that PilA from FeOB are able to form conductive pili similar to those in *Geobacter* spp., and this is consistent with the absence of the observation (e.g., via SEM or TEM) of conductive pili in FeOB.

### Alternative mechanisms

Notably, we did not find candidate Fe(II) oxidase genes in some FeOB, especially in more than half of the genomes of anaerobic nitrate-reducing FeOB, probably suggesting that their Fe(II) oxidases might be other types of redox proteins rather than Cyt *c* and MCO, or their Fe(II) oxidation mechanisms might be fundamentally different from the general models that we searched for. For example, Fe(II) oxidation may be achieved through chemical shuttles, result from abiotic oxidation by denitrification intermediates, or occur in the periplasm with reaction products being pumped to the outside, although these mechanisms may not be necessary for energy conservation via Fe(II) oxidation.

Rather than direct contact via a dedicated Fe(II) oxidase and conductive pili, cells may use electron shuttling chemicals such as flavins secreted by the cells (Okamoto et al., 2014) or humic acids which are naturally abundant in some environments (Lovley et al., 1998). However, outer membrane redox proteins (e.g., OmcA and MtrC) and/or the PCC system (MtrAB) are still required in order to exchange electrons between the cell membrane and extracellular chemical shuttles in *S. oneidensis* (Coursolle et al., 2010; Bucking et al., 2012; Okamoto et al., 2014).

Recently, a novel EET mechanism via shuttle chemicals was proposed for solid phase Fe(0) oxidation which involves redox enzymes released from lysed cells of *Methanococcus maripaludis* (Deutzmann et al., 2015). In this model, hydrogenase and formate dehydrogenase from lysed cells attach to the metal Fe(0) or cathode, and directly take electrons from the cathode in order to convert H^+^ to H_2_ and CO_2_ to formate. Bacteria then utilize the generated H_2_ and formate as electron donors, therefore indirectly obtaining electrons from Fe(0) via H_2_ and formate shuttles (Deutzmann et al., 2015). Theoretically, EET enzymes such as outer membrane redox proteins or PCC complex would not be necessary if cells use electrons from H_2_ and formate. In the case of Fe(0) oxidation, the redox potential for Fe(II)/Fe(0) is −0.47 V, lower than 2H^+^/H_2_ (−0.42 V) and CO_2_/formate (−0.42 V) at pH 7, and therefore these reactions are thermodynamically favorable, and the extracellular enzymes only serve as catalysts to accelerate the reactions. However, it is not likely that such a mechanism is used by neutrophilic FeOB, since Fe(OH)_3_/Fe(II) redox potential is higher, making the reactions to generate H_2_ and formate from Fe(II) thermodynamically unfavorable.

Notably, most nitrate-dependent FeOB can only grow in the presence of an organic co-substrate, therefore exhibiting heterotrophic/mixotrophic Fe(II) oxidation phenotypes. It is reasonable to assume that Fe(II) oxidation mechanisms in obligate lithoautotrophs could be very different from the mechanisms in heterotrophic/mixotrophic FeOB. For example, obligate chemolithoautotrophic FeOB obtain both reducing equivalents and energy exclusively from Fe(II) oxidation, whereas heterotrophic FeOB acquire reducing power and energy from the oxidation of organic carbon. Therefore, chemolithoautotrophic Fe(II) oxidation needs to be tightly coupled to inner membrane electron transferring processes, through which electrons from Fe(II) are fed into NADPH production and proton motive force-generating redox reactions. Therefore, for chemolithoautotrophs, Fe(II) oxidation cannot merely be a side reaction, such as in the case of Fe(II) detoxification or abiotic Fe(II) oxidation with denitrification intermediates. Coincidentally, putative EET genes were more frequently found in lithoautotrophic FeOB than in heterotrophic/mixotrophic FeOB, except for the putative PcoAB system, for which a role in metal detoxification is also possible as discussed earlier.

For nitrate-dependent FeOB, rather than extracellular oxidation, an alternative mechanism of Fe(II) oxidation was proposed (Carlson et al., 2013). This model suggests that nitrate-dependent Fe(II) oxidation is an innate capability of nitrate reducers, and a dedicated Fe(II) oxidoreductase is not required. Instead, oxidation of soluble Fe(II) in the periplasm is coupled to the reduction of nitrate or its reduction intermediates by directly donating electrons to the nitrate/nitrite/nitric oxide reductases and other periplasmic redox-active components, or the oxidation is abiotic using nitrite and nitric oxide as oxidants. With the increasing precipitation of Fe(III) in the periplasm, reductases are damaged, leaving nitrate-dependent Fe(II) oxidation dominated by abiotic reactions (Carlson et al., 2013). However, our genomic analysis alone cannot resolve whether such a mechanism is employed by the nitrate-reducing FeOB in their electron transferring reactions.

## Summary

In this study, we searched more than 70 FeOB genomes for candidate EET genes involved in neutrophilic Fe(II) oxidation. Overall, the results indicate their electron transferring pathways may be diverse, and one single EET mechanism or genetic system is not universally present in every FeOB. In addition to cataloging the presence or absence of current models of EET in Fe redox reactions, this study uncovered novel putative EET gene clusters (e.g., PCC3 and PCC4), thus extending our understanding of bacterial EET, and providing a list of interesting candidate genes for future research with transcriptomics, proteomics and physiological studies to further elucidate genes and pathways involved in electron transfer in microbial Fe(II) oxidation.

## Author contributions

ER and SH designed and initiated the study; SH analyzed the data, interpreted the results, and wrote the manuscript; ER, RB, and DE contributed to discussion of the results and revisions and improvement of the manuscript.

### Conflict of interest statement

The authors declare that the research was conducted in the absence of any commercial or financial relationships that could be construed as a potential conflict of interest.
