# Supplementary material for: Comparative Genomic Analysis of Neutrophilic Iron(II) Oxidizer Genomes for Candidate Genes in Extracellular Electron Transfer
Source: Front Microbiol. 2017 Aug 21;8:1584. doi: 10.3389/fmicb.2017.01584 (PMC5566968; doi:10.3389/fmicb.2017.01584)
Supplement: Supplementary file 1 [file Table1.DOCX]

**Supplementary Table 1. List of FeOB genomes included in the analysis**

| **IMG taxon ID** | **Genome name** | **Recovered genome size (bp)** | **Estimated genome completeness (%)** | **FeOB Group** | **Taxonomy -Class** | **Taxonomy -Order** | **Reference** |
| --- | --- | --- | --- | --- | --- | --- | --- |
| 643348541 | Acidovorax ebreus TPSY | 3796573 | 100 | Anaerobic | Betaproteobacteria | Burkholderiales | [Byrne-Bailey et al. (2010](#_ENREF_5)) |
| 645058786 | Acidovorax delafieldii 2AN | 4842094 | 98 | Anaerobic | Betaproteobacteria | Burkholderiales | [Chakraborty et al. (2011](#_ENREF_6)) |
| 2565956537 | Comamonadaceae sp. in NDFO enrichment | 3410349 | 95 | Anaerobic | Betaproteobacteria | Burkholderiales | [He et al. (2016](#_ENREF_17)) |
| 641522635 | Leptothrix cholodnii SP-6 | 4909403 | 100 | Microaerophilic | Betaproteobacteria | Burkholderiales | [Emerson and Ghiorse (1992](#_ENREF_10)) |
| 2506520002 | Leptothrix ochracea L12 | 875389 | 30 | Microaerophilic | Betaproteobacteria | Burkholderiales | [Fleming et al. (2011](#_ENREF_15)) |
| 2523533526 | Thiomonas sp. FB-6_DSM 25805 | 4279748 | 100 | Microaerophilic | Betaproteobacteria | Burkholderiales | [Fabisch et al. (2011](#_ENREF_13)) |
| 2574179766 | Thiomonas sp. FB-Cd_DSM 25617 | 4388462 | 100 | Microaerophilic | Betaproteobacteria | Burkholderiales | [Fabisch et al. (2011](#_ENREF_13)) |
| 2522125081 | Bordetella sp. FB-8_DSM 24873 | 4079718 | 100 | Microaerophilic | Betaproteobacteria | Burkholderiales | [Fabisch et al. (2011](#_ENREF_13)) |
| 2654587906 | Ralstonia sp. in Fe2S-oxidizing enrichment | 5658891 | 95 | Microaerophilic | Betaproteobacteria | Burkholderiales | [Percak-Dennett et al. (Submitted](#_ENREF_26)) |
| 2634166528 | Ralstonia sp. HM08-01 | 4787603 | 100 | Microaerophilic | Betaproteobacteria | Burkholderiales | [Swanner et al. (2011](#_ENREF_29)) |
| 2574179762 | Cupriavidus necator A5-1 | 9741376 | 99 | Microaerophilic/Anaerobic | Betaproteobacteria | Burkholderiales | [Benzine et al. (2013](#_ENREF_3)) |
| 2528768215 | Dechloromonas agitata is5 | 3627226 | 100 | Anaerobic | Betaproteobacteria | Rhodocyclales | [Benzine et al. (2013](#_ENREF_3)) |
| 637000088 | Dechloromonas aromatica RCB | 4501104 | 100 | Anaerobic | Betaproteobacteria | Rhodocyclales | [Dubinina and Sorokina (2014](#_ENREF_7)) |
| 2508501046 | Azospira suillum PS | 3806980 | 100 | Anaerobic | Betaproteobacteria | Rhodocyclales | [Lack et al. (2002](#_ENREF_25)) |
| 2264867016 | Gallionella sp. SCGC AAA018-N21 | 1396108 | 46 | Microaerophilic | Betaproteobacteria | Gallionellales | [Emerson (2014](#_ENREF_9)) |
| 648028028 | Gallionella capsiferriformans ES-2 | 3162471 | 99 | Microaerophilic | Betaproteobacteria | Gallionellales | [Emerson and Moyer (1997](#_ENREF_11)) |
| 2565956535 | Gallionellaceae sp. in NDFO enrichment | 2644639 | 98 | Anaerobic | Betaproteobacteria | Gallionellales | [He et al. (2016](#_ENREF_17)) |
| 2645727616 | Ferriphaselus amnicola OYT1 | 2684190 | 98 | Microaerophilic | Betaproteobacteria | Gallionellales | [Kato et al. (2015](#_ENREF_21)) |
| 2574180436 | Ferriphaselus sp. R-1 | 2441599 | 98 | Microaerophilic | Betaproteobacteria | Gallionellales | [Krepski et al. (2012](#_ENREF_23)) |
| 646564569 | Sideroxydans lithotrophicus ES-1 | 3003656 | 100 | Microaerophilic | Betaproteobacteria | Gallionellales | [Emerson and Moyer (1997](#_ENREF_11)) |
| 643886068 | Pseudogulbenkiania ferrooxidans 2002 | 4228787 | 100 | Anaerobic | Betaproteobacteria | Neisseriales | [Weber et al. (2006](#_ENREF_31)) |
| 637000324 | Thiobacillus denitrificans ATCC 25259 | 2909809 | 100 | Anaerobic | Betaproteobacteria | Hydrogenophilales | [Beller et al. (2013](#_ENREF_2)) |
| 650377991 | Marinobacter adhaerens HP15 | 4651725 | 100 | Microaerophilic | Gammaproteobacteria | Alteromonadales | [Ullrich (2011](#_ENREF_30)) |
| 2648501751 | Marinobacter subterrani JG233 | 4453613 | 100 | Microaerophilic | Gammaproteobacteria | Alteromonadales | [Bonis and Gralnick (2015](#_ENREF_4)) |
| 2540341173 | Marinobacter hydrocarbonoclasticus ATCC 49840 | 3989480 | 100 | Microaerophilic | Gammaproteobacteria | Alteromonadales | [Barbe (2013](#_ENREF_1)) |
| 639633037 | Marinobacter aquaeolei VT8 | 4779762 | 100 | Microaerophilic | Gammaproteobacteria | Alteromonadales | [Singer et al. (2011](#_ENREF_28)) |
| 2545824694 | Marinobacter santoriniensis NKSG1 | 4033468 | 100 | Anaerobic | Gammaproteobacteria | Alteromonadales | [Handley et al. (2009](#_ENREF_16)) |
| 2565956540 | Rhodanobacter sp. in NDFO enrichment | 3256089 | 84 | Anaerobic | Gammaproteobacteria | Xanthomonadales | [He et al. (2016](#_ENREF_17)) |
| 2513237158 | Mariprofundus ferrooxydans M34 | 2736181 | 100 | Microaerophilic | Zetaproteobacteria | Mariprofundales | [Field et al. (2015](#_ENREF_14)) |
| 2648501925 | Mariprofundus ferrooxydans JV-1 | 2850202 | 100 | Microaerophilic | Zetaproteobacteria | Mariprofundales | [Emerson and Moyer (2002](#_ENREF_12)) |
| 639857004 | Mariprofundus ferrooxydans PV-1 | 2867087 | 100 | Microaerophilic | Zetaproteobacteria | Mariprofundales | [Emerson and Moyer (2002](#_ENREF_12)) |
| 2571042360 | Mariprofundus sp. EKF-M39 | 2718232 | 98 | Microaerophilic | Zetaproteobacteria | Mariprofundales | [Field et al. (2015](#_ENREF_14)) |
| 2558860158 | Zetaproteobacterium SCGC AB-706-D06 | 437623 | 18 | Microaerophilic | Zetaproteobacteria | unclassified | [Field et al. (2015](#_ENREF_14)) |
| 2524614791 | Zetaproteobacterium SCGC AB-602_L11 | 327729 | 5 | Microaerophilic | Zetaproteobacteria | unclassified | [Field et al. (2015](#_ENREF_14)) |
| 2524614790 | Zetaproteobacterium SCGC AB-137_M18 | 450945 | 9 | Microaerophilic | Zetaproteobacteria | unclassified | [Field et al. (2015](#_ENREF_14)) |
| 2264867013 | Zetaproteobacterium SCGC AB-133-G06 | 1091316 | 43 | Microaerophilic | Zetaproteobacteria | unclassified | [Field et al. (2015](#_ENREF_14)) |
| 2264867008 | Zetaproteobacterium SCGC AB-602-C20 | 990274 | 23 | Microaerophilic | Zetaproteobacteria | unclassified | [Field et al. (2015](#_ENREF_14)) |
| 2524614788 | Zetaproteobacterium SCGC AB-133_D10 | 1566037 | 49 | Microaerophilic | Zetaproteobacteria | unclassified | [Field et al. (2015](#_ENREF_14)) |
| 2264867010 | Zetaproteobacterium SCGC AB-137-J06 | 889605 | 32 | Microaerophilic | Zetaproteobacteria | unclassified | [Field et al. (2015](#_ENREF_14)) |
| 2265123003 | Zetaproteobacterium SCGC AB-137-I08 | 2048981 | 70 | Microaerophilic | Zetaproteobacteria | unclassified | [Field et al. (2015](#_ENREF_14)) |
| 2524614781 | Zetaproteobacterium SCGC AB-602_F03 | 990942 | 41 | Microaerophilic | Zetaproteobacteria | unclassified | [Field et al. (2015](#_ENREF_14)) |
| 2524614793 | Zetaproteobacterium SCGC AB-133_M17 | 1492986 | 57 | Microaerophilic | Zetaproteobacteria | unclassified | [Field et al. (2015](#_ENREF_14)) |
| 2524614792 | Zetaproteobacterium SCGC AB-604_P22 | 605565 | 36 | Microaerophilic | Zetaproteobacteria | unclassified | [Field et al. (2015](#_ENREF_14)) |
| 2524614794 | Zetaproteobacterium SCGC AB-137_G16 | 891863 | 44 | Microaerophilic | Zetaproteobacteria | unclassified | [Field et al. (2015](#_ENREF_14)) |
| 2264867014 | Zetaproteobacterium SCGC AB-133-C04 | 1323771 | 62 | Microaerophilic | Zetaproteobacteria | unclassified | [Field et al. (2015](#_ENREF_14)) |
| 2558860157 | Zetaproteobacterium SCGC AB-706-B05 | 1054783 | 43 | Microaerophilic | Zetaproteobacteria | unclassified | [Field et al. (2015](#_ENREF_14)) |
| 2528768164 | Zetaproteobacterium SCGC AB-137-L23 | 1695012 | 64 | Microaerophilic | Zetaproteobacteria | unclassified | [Field et al. (2015](#_ENREF_14)) |
| 2524614796 | Zetaproteobacterium SCGC AB-137_C09B | 2348799 | 84 | Microaerophilic | Zetaproteobacteria | unclassified | [Field et al. (2015](#_ENREF_14)) |
| 2264867009 | Zetaproteobacterium SCGC AB-137-C09 | 2446432 | 83 | Microaerophilic | Zetaproteobacteria | unclassified | [Field et al. (2015](#_ENREF_14)) |
| 2264867015 | Zetaproteobacterium SCGC AB-602-E04 | 452710 | 21 | Microaerophilic | Zetaproteobacteria | unclassified | [Field et al. (2015](#_ENREF_14)) |
| 2528768165 | Zetaproteobacterium SCGC AC-673-N02 | 950282 | 8 | Microaerophilic | Zetaproteobacteria | unclassified | [Field et al. (2015](#_ENREF_14)) |
| 2528768224 | Zetaproteobacterium SCGC AC-673-B17 | 1333296 | 34 | Microaerophilic | Zetaproteobacteria | unclassified | [Field et al. (2015](#_ENREF_14)) |
| 2264867011 | Zetaproteobacterium SCGC AB-604-B04 | 947176 | 31 | Microaerophilic | Zetaproteobacteria | unclassified | [Field et al. (2015](#_ENREF_14)) |
| 2524614605 | Zetaproteobacterium SCGC AC-673-M07 | 1484115 | 42 | Microaerophilic | Zetaproteobacteria | unclassified | [Field et al. (2015](#_ENREF_14)) |
| 2524614795 | Zetaproteobacterium SCGC AB-604_O11 | 665360 | 15 | Microaerophilic | Zetaproteobacteria | unclassified | [Field et al. (2015](#_ENREF_14)) |
| 2528768162 | Zetaproteobacterium SCGC AC-673-C02 | 878954 | 27 | Microaerophilic | Zetaproteobacteria | unclassified | [Field et al. (2015](#_ENREF_14)) |
| 2264867012 | Zetaproteobacterium SCGC AB-604-O16 | 979689 | 38 | Microaerophilic | Zetaproteobacteria | unclassified | [Field et al. (2015](#_ENREF_14)) |
| 2528768166 | Zetaproteobacterium SCGC AC-675-C07 | 618800 | 23 | Microaerophilic | Zetaproteobacteria | unclassified | [Field et al. (2015](#_ENREF_14)) |
| 2524614546 | Bradyrhizobium japonicum in8p8 | 7589915 | 100 | Anaerobic | Alphaproteobacteria | Rhizobiales | [Benzine et al. (2013](#_ENREF_3)) |
| 2524614545 | Bradyrhizobium japonicum is5 | 7588794 | 100 | Microaerophilic/Anaerobic | Alphaproteobacteria | Rhizobiales | [Benzine et al. (2013](#_ENREF_3)) |
| 2529292555 | Bradyrhizobium japonicum 22 | 7504559 | 100 | Microaerophilic/Anaerobic | Alphaproteobacteria | Rhizobiales | [Benzine et al. (2013](#_ENREF_3)) |
| 2565956536 | Bradyrhizobium sp. in NDFO enrichment | 7164947 | 99 | Anaerobic | Alphaproteobacteria | Rhizobiales | [He et al. (2016](#_ENREF_17)) |
| 642555153 | Rhodopseudomonas palustris TIE-1 | 5744041 | 100 | Phototrophic | Alphaproteobacteria | Rhizobiales | [Jiao et al. (2005](#_ENREF_20)) |
| 2654587905 | Bradyrhizobium sp. in Fe2S-oxidizing enrichment | 6524326 | 98 | Microaerophilic | Alphaproteobacteria | Rhizobiales | [Percak-Dennett et al., Submitted](#_ENREF_8)) |
| 2654587907 | Hyphomicrobium sp. in Fe2S-oxidizing enrichment | 4553600 | 91 | Microaerophilic | Alphaproteobacteria | Rhizobiales | [Percak-Dennett et al., Submitted](#_ENREF_8)) |
| 649633090 | Rhodomicrobium vannielii ATCC 17100 | 4014469 | 98 | Phototrophic | Alphaproteobacteria | Rhizobiales | [Heising and Schink (1998](#_ENREF_18)) |
| 2508501030 | Paracoccus pantotrophus J46 | 4658858 | 99 | Anaerobic (with nitrite) | Alphaproteobacteria | Rhodobacterales | [Kumaraswamy et al. (2006](#_ENREF_24)) |
| 2508501113 | Paracoccus pantotrophus J40 | 4244593 | 99 | Anaerobic (with nitrite) | Alphaproteobacteria | Rhodobacterales | [Kumaraswamy et al. (2006](#_ENREF_24)) |
| 2615840723 | Paracoccus versutus DSM 582 | 5627664 | 99 | Anaerobic (with nitrite) | Alphaproteobacteria | Rhodobacterales | [Kumaraswamy et al. (2006](#_ENREF_24)) |
| 639633048 | Paracoccus denitrificans PD1222 | 5236194 | 99 | Anaerobic | Alphaproteobacteria | Rhodobacterales | [Klueglein et al. (2014](#_ENREF_22)) |
| 646564563 | Rhodobacter capsulatus SB1003 | 3871920 | 99 | Phototrophic | Alphaproteobacteria | Rhodobacterales | [Poulain and Newman (2009](#_ENREF_27)) |
| 647000304 | Rhodobacter sp. SW2 | 3514342 | 96 | Phototrophic | Alphaproteobacteria | Rhodobacterales | [Ehrenreich and Widdel (1994](#_ENREF_8)) |
| 2565956539 | Rhizobium bin in NDFO enrichment | 4909323 | 70 | Anaerobic | Alphaproteobacteria | Rhizobiales | [He et al. (2016](#_ENREF_17)) |
| 638341060 | Chlorobium ferrooxidans DSM 13031 | 2538957 | 95 | Phototrophic | Chlorobia | Chlorobiales | [Heising et al. (1999](#_ENREF_19)) |

**Supplementary References:**

Barbe, V. (2013). Marinobacter hydrocarbonoclasticus ATCC 49840. URL https://img.jgi.doe.gov/cgi-bin/m/main.cgi?section=TaxonDetail&page=taxonDetail&taxon_oid=2540341173

Beller, H.R., Zhou, P., Legler, T.C., Chakicherla, A., Kane, S., Letain, T.E., and P, A.O.D. (2013) Genome-enabled studies of anaerobic, nitrate-dependent iron oxidation in the chemolithoautotrophic bacterium Thiobacillus denitrificans. *Front Microbiol* **4**: 249.

Benzine, J., Shelobolina, E., Xiong, M.Y., Kennedy, D.W., McKinley, J.P., Lin, X., and Roden, E.E. (2013) Fe-phyllosilicate redox cycling organisms from a redox transition zone in Hanford 300 Area sediments. *Frontiers in Microbiology* **4**: 388.

Bonis, B.M., and Gralnick, J.A. (2015) Marinobacter subterrani, a genetically tractable neutrophilic Fe(II)-oxidizing strain isolated from the Soudan Iron Mine. *Frontiers in Microbiology* **6**: 719.

Byrne-Bailey, K.G., Weber, K.A., Chair, A.H., Bose, S., Knox, T., Spanbauer, T.L. et al. (2010) Completed genome sequence of the anaerobic iron-oxidizing bacterium *Acidovorax ebreus* strain TPSY. *J Bacteriol* **192**: 1475-1476.

Chakraborty, A., Roden, E.E., Schieber, J., and Picardal, F. (2011) Enhanced growth of Acidovorax sp. strain 2AN during nitrate-dependent Fe(II) oxidation in batch and continuous-flow systems. *Appl Environ Microbiol* **77**: 8548-8556.

Dubinina, G.A., and Sorokina, A.Y. (2014) Neutrophilic lithotrophic iron-oxidizing prokaryotes and their role in the biogeochemical processes of the iron cycle. *Microbiology* **83**: 1-14.

Ehrenreich, A., and Widdel, F. (1994) Anaerobic oxidation of ferrous iron by purple bacteria, a new type of phototrophic metabolism. *Applied and Environmental Microbiology* **60**: 4517-4526.

Emerson, D. (2014). Single cell genome sequencing of biomineralizing bacteria - Gallionella sp. SCGC AAA018-N21. URL https://img.jgi.doe.gov/cgi-bin/pub/main.cgi?section=TaxonDetail&page=taxonDetail&taxon_oid=2264867016

Emerson, D., and Ghiorse, W.C. (1992) Isolation, Cultural Maintenance, and Taxonomy of a Sheath-Forming Strain of Leptothrix discophora and Characterization of Manganese-Oxidizing Activity Associated with the Sheath. *Appl Environ Microbiol* **58**: 4001-4010.

Emerson, D., and Moyer, C. (1997) Isolation and characterization of novel iron-oxidizing bacteria that grow at circumneutral pH. *Appl Environ Microbiol* **63**: 4784-4792.

Emerson, D., and Moyer, C.L. (2002) Neutrophilic Fe-Oxidizing Bacteria Are Abundant at the Loihi Seamount Hydrothermal Vents and Play a Major Role in Fe Oxide Deposition. *Applied and Environmental Microbiology* **68**: 3085-3093.

Fabisch, M., Beulig, F., Akob, D.M., and Kuesel, K. (2011) New Thiomonas and Bordetella strains involved in iron oxidation at a slightly acidic, heavy metal contaminated creek. In *Goldschmidt Conference*. Prague, Czech Republic.

Field, E.K., Sczyrba, A., Lyman, A.E., Harris, C.C., Woyke, T., Stepanauskas, R., and Emerson, D. (2015) Genomic insights into the uncultivated marine Zetaproteobacteria at Loihi Seamount. *ISME J* **9**: 857-870.

Fleming, E.J., Langdon, A.E., Martinez-Garcia, M., Stepanauskas, R., Poulton, N.J., Masland, E.D., and Emerson, D. (2011) What's new is old: resolving the identity of Leptothrix ochracea using single cell genomics, pyrosequencing and FISH. *PLoS One* **6**: e17769.

Handley, K.M., Héry, M., and Lloyd, J.R. (2009) Marinobacter santoriniensis sp. nov., an arsenate-respiring and arsenite-oxidizing bacterium isolated from hydrothermal sediment. *International Journal of Systematic and Evolutionary Microbiology* **59**: 886-892.

He, S., Tominski, C., Kappler, A., Behrens, S., and Roden, E.E. (2016) Metagenomic Analyses of the Autotrophic Fe(II)-Oxidizing, Nitrate-Reducing Enrichment Culture KS. *Appl Environ Microbiol* **82**: 2656-2668.

Heising, S., and Schink, B. (1998) Phototrophic oxidation of ferrous iron by a Rhodomicrobium vannielii strain. *Microbiology* **144 ( Pt 8)**: 2263-2269.

Heising, S., Richter, L., Ludwig, W., and Schink, B. (1999) Chlorobium ferrooxidans sp. nov., a phototrophic green sulfur bacterium that oxidizes ferrous iron in coculture with a "Geospirillum" sp. strain. *Arch Microbiol* **172**: 116-124.

Jiao, Y., Kappler, A., Croal, L.R., and Newman, D.K. (2005) Isolation and characterization of a genetically tractable photoautotrophic Fe(II)-oxidizing bacterium, Rhodopseudomonas palustris strain TIE-1. *Appl Environ Microbiol* **71**: 4487-4496.

Kato, S., Ohkuma, M., Powell, D.H., Krepski, S.T., Oshima, K., Hattori, M. et al. (2015) Comparative genomic insights into ecophysiology of neutrophilic, microaerophilic iron oxidizing bacteria. *Front Microbiol* **6**: 1265.

Klueglein, N., Zeitvogel, F., Stierhof, Y.-D., Floetenmeyer, M., Konhauser, K.O., Kappler, A., and Obst, M. (2014) Potential role of nitrite for abiotic Fe(II) oxidation and cell encrustation during nitrate reduction by denitrifying bacteria. *Appl Environ Microbiol* **80**: 1051-1061.

Krepski, S.T., Hanson, T.E., and Chan, C.S. (2012) Isolation and characterization of a novel biomineral stalk-forming iron-oxidizing bacterium from a circumneutral groundwater seep. *Environ Microbiol* **14**: 1671-1680.

Kumaraswamy, R., Sjollema, K., Kuenen, G., van Loosdrecht, M., and Muyzer, G. (2006) Nitrate-dependent [Fe(II)EDTA]2- oxidation by Paracoccus ferrooxidans sp. nov., isolated from a denitrifying bioreactor. *Syst Appl Microbiol* **29**: 276-286.

Lack, J.G., Chaudhuri, S.K., Chakraborty, R., Achenbach, L.A., and Coates, J.D. (2002) Anaerobic Biooxidation of Fe(II) by Dechlorosoma suillum. *Microbial Ecology* **43**: 424-431.

Percak-Dennett, E., He, S., Converse, B., Konishi, H., Xu, H., Corcoran, A. et al. (Submitted) Aerobic microbial pyrite oxidation at circumneutral pH.

Poulain, A.J., and Newman, D.K. (2009) Rhodobacter capsulatus Catalyzes Light-Dependent Fe(II) Oxidation under Anaerobic Conditions as a Potential Detoxification Mechanism. *Applied and Environmental Microbiology* **75**: 6639-6646.

Singer, E., Webb, E.A., Nelson, W.C., Heidelberg, J.F., Ivanova, N., Pati, A., and Edwards, K.J. (2011) Genomic Potential of Marinobacter aquaeolei, a Biogeochemical “Opportunitroph”. *Applied and Environmental Microbiology* **77**: 2763-2771.

Swanner, E.D., Nell, R.M., and Templeton, A.S. (2011) Ralstonia species mediate Fe-oxidation in circumneutral, metal-rich subsurface fluids of Henderson mine, CO. *Chemical Geology* **284**: 339-350.

Ullrich, M.S. (2011). Marinobacter adhaerens HP15. URL https://img.jgi.doe.gov/cgi-bin/m/main.cgi?section=TaxonDetail&page=taxonDetail&taxon_oid=650377991

Weber, K.A., Pollock, J., Cole, K.A., O'Connor, S.M., Achenbach, L.A., and Coates, J.D. (2006) Anaerobic nitrate-dependent iron(II) bio-oxidation by a novel lithoautotrophic betaproteobacterium, strain 2002. *Appl Environ Microbiol* **72**: 686-694.
