# Supplementary material for: Comparative Genomic Analysis of Neutrophilic Iron(II) Oxidizer Genomes for Candidate Genes in Extracellular Electron Transfer
Source: Front Microbiol. 2017 Aug 21;8:1584. doi: 10.3389/fmicb.2017.01584 (PMC5566968; doi:10.3389/fmicb.2017.01584)
Supplement: Supplementary file 2 [file Table2.DOCX]

**Supplementary Table 2. Proteins homologous to *A. ferrooxydans* Cyc2 from bacteria not known for Fe(II) oxidation**

| **IMG Gene ID** | **Protein length (aa)** | **Percent Identity (%)** | **E-value** | **Bit Score** | **Genome Name** | **Phylum** |
| --- | --- | --- | --- | --- | --- | --- |
| 2504619835 | 449 | 32.69 | 1.90E-59 | 229 | Flavobacterium sp. GOBB3-C103-3 | Bacteroidetes |
| 2723877579 | 446 | 35.03 | 6.20E-56 | 218 | Pandoraea thiooxydans ATSB16 | Proteobacteria |
| 2649142128 | 446 | 35.03 | 6.20E-56 | 218 | Pandoraea thiooxydans DSM 25325 | Proteobacteria |
| 2712202630 | 446 | 34.07 | 2.10E-54 | 213 | Ralstonia pickettii SSH4 | Proteobacteria |
| 2676342706 | 465 | 34.09 | 2.00E-53 | 210 | Methylocapsa palsarum NE2 | Proteobacteria |
| 2636887052 | 446 | 33.75 | 3.90E-53 | 209 | Burkholderia vietnamiensis LMG 10929 | Proteobacteria |
| 2516190291 | 437 | 30.43 | 1.40E-52 | 208 | Methylomonas sp. 11b | Proteobacteria |
| 2695598091 | 446 | 33.68 | 2.60E-52 | 207 | Ralstonia solanacearum IBSBF1900 | Proteobacteria |
| 2678161134 | 446 | 33.68 | 2.60E-52 | 207 | Ralstonia solanacearum P597 | Proteobacteria |
| 2655899363 | 446 | 33.68 | 2.60E-52 | 207 | Ralstonia solanacearum B50 | Proteobacteria |
| 2523628613 | 447 | 33.61 | 4.30E-52 | 206 | Ralstonia sp. AU12-08 (IVD) | Proteobacteria |
| 2632161144 | 439 | 31.95 | 4.30E-52 | 206 | Chromobacterium vaccinii MWU205 | Proteobacteria |
| 2629458485 | 439 | 31.95 | 4.30E-52 | 206 | Chromobacterium vaccinii MWU328 | Proteobacteria |
| 2570846703 | 446 | 34.07 | 1.10E-51 | 205 | Ralstonia solanacearum P673 | Proteobacteria |
| 2714096225 | 439 | 32.43 | 1.10E-51 | 205 | Chromobacterium subtsugae MWU2920 | Proteobacteria |
| 2695578753 | 446 | 33.68 | 1.10E-51 | 205 | Ralstonia solanacearum UW181 | Proteobacteria |
| 2655877439 | 446 | 33.68 | 1.10E-51 | 205 | Ralstonia solanacearum Grenada 9-1 | Proteobacteria |
| 2562607275 | 446 | 33.68 | 1.10E-51 | 205 | Ralstonia solanacearum CFBP2957 | Proteobacteria |
| 2721279830 | 439 | 31.74 | 1.80E-51 | 204 | Chromobacterium vaccinii 21-1 | Proteobacteria |
| 2695619073 | 446 | 33.47 | 4.30E-51 | 203 | Ralstonia solanacearum CFBP7014 | Proteobacteria |
| 2628330780 | 439 | 32.35 | 4.30E-51 | 203 | Chromobacterium piscinae ND17 | Proteobacteria |
| 2600812805 | 446 | 33.97 | 8.10E-51 | 202 | Paraburkholderia kururiensis thiooxydans NBRC 107107 | Proteobacteria |
| 2631528143 | 446 | 32.99 | 8.10E-51 | 202 | Burkholderia pseudomallei ABCPW 111 | Proteobacteria |
| 2597913977 | 490 | 31.65 | 8.10E-51 | 202 | Bradyrhizobium sp. STM 3843 | Proteobacteria |
| 2636923465 | 446 | 33.4 | 1.20E-50 | 201 | Burkholderia thailandensis E254 | Proteobacteria |
| 2635808557 | 446 | 33.4 | 1.20E-50 | 201 | Burkholderia thailandensis MSMB59 | Proteobacteria |
| 2633799177 | 446 | 33.4 | 1.20E-50 | 201 | Burkholderia thailandensis 2002721643 | Proteobacteria |
| 2632122048 | 446 | 33.4 | 1.20E-50 | 201 | Burkholderia thailandensis 34 | Proteobacteria |
| 2630678211 | 446 | 33.4 | 1.20E-50 | 201 | Burkholderia thailandensis Phuket 4W-1 | Proteobacteria |
| 2630092482 | 446 | 33.4 | 1.20E-50 | 201 | Burkholderia thailandensis BDK | Proteobacteria |
| 2585941208 | 446 | 33.4 | 1.20E-50 | 201 | Burkholderia thailandensis USAMRU Malaysia #20 | Proteobacteria |
| 2559273074 | 446 | 33.4 | 1.20E-50 | 201 | Burkholderia thailandensis E444 | Proteobacteria |
| 2559268833 | 446 | 33.4 | 1.20E-50 | 201 | Burkholderia thailandensis 2002721723 | Proteobacteria |
| 2559259245 | 446 | 33.4 | 1.20E-50 | 201 | Burkholderia thailandensis H0587 | Proteobacteria |
| 2547999166 | 446 | 33.4 | 1.20E-50 | 201 | Burkholderia thailandensis E355 | Proteobacteria |
| 637839079 | 446 | 33.4 | 1.20E-50 | 201 | Burkholderia thailandensis E264, ATCC 700388 | Proteobacteria |
| 2718826513 | 446 | 32.78 | 7.60E-50 | 199 | Ralstonia solanacearum FJAT-1458 | Proteobacteria |
| 2598129562 | 446 | 32.78 | 7.60E-50 | 199 | Ralstonia solanacearum Rs-09-161 | Proteobacteria |
| 2554992125 | 446 | 32.78 | 7.60E-50 | 199 | Ralstonia solanacearum SD54 | Proteobacteria |
| 2549746721 | 446 | 32.78 | 7.60E-50 | 199 | Ralstonia solanacearum FJAT-1458 | Proteobacteria |
| 2549741631 | 446 | 32.78 | 7.60E-50 | 199 | Ralstonia solanacearum FJAT-91 | Proteobacteria |
| 2628342326 | 446 | 33.2 | 7.60E-50 | 199 | Burkholderia oklahomensis C6786 | Proteobacteria |
| 2598404916 | 446 | 33.2 | 7.60E-50 | 199 | Burkholderia oklahomensis BDU | Proteobacteria |
| 2621211688 | 453 | 30.37 | 1.10E-49 | 198 | Thioploca ingrica | Proteobacteria |
| 2716757859 | 461 | 31.74 | 2.60E-49 | 197 | Burkholderia sp. CF145 | Proteobacteria |
| 2657096016 | 461 | 31.19 | 1.20E-48 | 195 | Paraburkholderia hospita LMG 20598 | Proteobacteria |
| 641923098 | 428 | 33.26 | 1.20E-48 | 195 | Burkholderia oklahomensis C6786 | Proteobacteria |
| 641918692 | 428 | 33.26 | 1.20E-48 | 195 | Burkholderia oklahomensis EO147 | Proteobacteria |
| 2597911083 | 514 | 31.75 | 6.20E-48 | 193 | Bradyrhizobium sp. STM 3843 | Proteobacteria |
| 2538731527 | 462 | 31.33 | 1.40E-47 | 192 | Paraburkholderia terrae BS001 | Proteobacteria |
| 2714105722 | 439 | 30.43 | 2.30E-47 | 191 | Chromobacterium subtsugae MWU2387 | Proteobacteria |
| 2616359284 | 461 | 31.33 | 2.30E-47 | 191 | Burkholderia sp. YR281 | Proteobacteria |
| 2596953190 | 437 | 32.03 | 1.20E-46 | 189 | Massilia flava CGMCC 1.10685 | Proteobacteria |
| 2514548088 | 451 | 30.39 | 1.70E-46 | 188 | Cupriavidus basilensis OR16 | Proteobacteria |
| 2578453291 | 458 | 32.22 | 3.20E-46 | 187 | Dyella jiangningensis SBZ 3-12 | Proteobacteria |
| 2636530532 | 517 | 31.23 | 6.10E-46 | 186 | Bradyrhizobium sp. SEMIA 6208 | Proteobacteria |
| 2536148201 | 448 | 30.98 | 1.60E-45 | 185 | Burkholderia sp. TJI49 | Proteobacteria |
| 2630400743 | 461 | 30.83 | 3.50E-45 | 184 | Burkholderia sp. MSHR3999 | Proteobacteria |
| 2510255561 | 485 | 31.28 | 3.50E-45 | 184 | Methylocapsa acidiphila B2 | Proteobacteria |
| 2628969672 | 461 | 30.83 | 7.60E-45 | 183 | Burkholderia ubonensis MSMB22 | Proteobacteria |
| 2517553761 | 464 | 30.74 | 3.20E-44 | 181 | Methylosarcina fibrata AML-C10 | Proteobacteria |
| 2726876947 | 457 | 31.88 | 3.20E-44 | 181 | Burkholderia concitans LMG 29315 | Proteobacteria |
| 2630047613 | 457 | 31.88 | 3.20E-44 | 181 | Burkholderia sp. MR1 | Proteobacteria |
| 2598127413 | 450 | 30.49 | 6.00E-44 | 180 | Ralstonia solanacearum Rs-09-161 | Proteobacteria |
| 2634941930 | 464 | 30.21 | 6.00E-44 | 180 | Methylosarcina lacus-69 (UID4274) | Proteobacteria |
| 2598220127 | 450 | 30.66 | 1.60E-43 | 179 | Ralstonia solanacearum Rs-10-244 | Proteobacteria |
| 2517249728 | 464 | 30.21 | 1.60E-43 | 179 | Methylosarcina lacus LW14 | Proteobacteria |
| 2700928529 | 517 | 30.64 | 1.60E-43 | 179 | Bradyrhizobium erythrophlei GAS478 | Proteobacteria |
| 2634852957 | 476 | 30.04 | 5.50E-43 | 177 | Opitutae-129 (UID2982) | Verrucomicrobia |
| 2619988218 | 464 | 32.22 | 5.50E-43 | 177 | Dechloromonas sp. EBPR_Bin_104 | Proteobacteria |
| 2609799710 | 450 | 30.6 | 5.50E-43 | 177 | Collimonas sp. OK307 | Proteobacteria |
| 2653887682 | 518 | 31.15 | 5.50E-43 | 177 | Bradyrhizobium sp. Gha | Proteobacteria |
| 2652266820 | 450 | 30.44 | 5.50E-43 | 177 | Ralstonia solanacearum YC45 | Proteobacteria |
| 2562603809 | 456 | 30.49 | 5.50E-43 | 177 | Ralstonia solanacearum CMR15 | Proteobacteria |
| 2551920040 | 451 | 30.79 | 1.00E-42 | 176 | Cupriavidus basilensis B-8 | Proteobacteria |
| 651002515 | 452 | 31.52 | 1.00E-42 | 176 | Nitrosomonas sp. IS79A3 | Proteobacteria |
| 2547402577 | 450 | 30.12 | 2.20E-42 | 175 | Ralstonia solanacearum Y45 | Proteobacteria |
| 2651157958 | 450 | 30.12 | 2.20E-42 | 175 | Ralstonia solanacearum Rs-T02 | Proteobacteria |
| 2700926766 | 517 | 30.36 | 5.70E-42 | 174 | Bradyrhizobium erythrophlei GAS478 | Proteobacteria |
| 2501411770 | 473 | 30.11 | 7.80E-42 | 173 | Paraburkholderia silvatlantica SRMrh-20 | Proteobacteria |
| 2511100771 | 466 | 30.11 | 7.80E-42 | 173 | Paraburkholderia silvatlantica SRMrh-20 | Proteobacteria |
| 2685829211 | 469 | 31.52 | 1.50E-41 | 172 | Rhodopila sp. LVNP | Proteobacteria |
| 2723084434 | 406 | 31.58 | 3.60E-41 | 171 | Ralstonia solanacearum EP1 | Proteobacteria |
| 2709537374 | 423 | 30.92 | 3.60E-41 | 171 | Gammaproteobacteria bacterium RBG_16_57_12 | Proteobacteria |
| 2511107514 | 466 | 30.11 | 3.60E-41 | 171 | Burkholderia silvatlantica PVA5 (IHQD assembly) | Proteobacteria |
| 2636913632 | 453 | 30.33 | 3.60E-41 | 171 | Nitrosospira lacus APG3 | Proteobacteria |
| 2501071901 | 473 | 30.11 | 3.60E-41 | 171 | Burkholderia silvatlantica PVA5 | Proteobacteria |
| 2511632122 | 442 | 30.26 | 8.40E-41 | 170 | Pseudogulbenkiania sp. NH8B | Proteobacteria |
| 2521976332 | 461 | 30.3 | 8.40E-41 | 170 | Burkholderia cepacia GG4 | Proteobacteria |
| 2714739554 | 435 | 30.02 | 2.90E-40 | 168 | Massilia sp. Root335 | Proteobacteria |
| 2671445702 | 455 | 30.08 | 2.90E-40 | 168 | Nitrosospira multiformis Nl4 | Proteobacteria |
| 2510415289 | 461 | 30.45 | 3.10E-39 | 165 | Rhodanobacter sp. OR87 | Proteobacteria |
| 2510323028 | 461 | 30.45 | 6.70E-39 | 164 | Rhodanobacter sp. OR92 | Proteobacteria |
| 2585741477 | 461 | 30.39 | 1.10E-38 | 163 | Rhodanobacter sp. FW104-T7 (Draft Assembly) | Proteobacteria |
| 2585895491 | 450 | 30.02 | 2.00E-38 | 162 | Methylotenera sp. G11 | Proteobacteria |
| 2553029806 | 461 | 30.53 | 2.00E-38 | 162 | Dyella ginsengisoli LA-4 | Proteobacteria |
| 649772731 | 450 | 30.04 | 3.70E-38 | 161 | Methylovorus sp. MP688 | Proteobacteria |
| 2574134454 | 455 | 30.04 | 1.10E-37 | 160 | Methylomonas | Proteobacteria |
| 2515877853 | 454 | 30.88 | 5.60E-36 | 154 | Uliginosibacterium gangwonense DSM 18521 | Proteobacteria |
| 2523410377 | 454 | 30.71 | 1.30E-35 | 153 | Nevskia ramosa DSM 11499 | Proteobacteria |
| 648843130 | 460 | 30.15 | 3.60E-33 | 145 | Methylobacter tundripaludum SV96 | Proteobacteria |
| 2727726885 | 448 | 30.37 | 3.60E-33 | 145 | Nitrosomonas sp. Nm120 | Proteobacteria |
| 2517428901 | 460 | 30.04 | 7.30E-33 | 144 | Methylobacter luteus IMV-B-3098 | Proteobacteria |
