# Supplementary material for: Comparative Genomic Analysis of Neutrophilic Iron(II) Oxidizer Genomes for Candidate Genes in Extracellular Electron Transfer
Source: Front Microbiol. 2017 Aug 21;8:1584. doi: 10.3389/fmicb.2017.01584 (PMC5566968; doi:10.3389/fmicb.2017.01584)
Supplement: Supplementary file 3 [file Table3.DOCX]

**Supplementary Table 3. Proteobacterial class, physiology and isolation source of the novel PCC3-containing genomes*^a,b^***


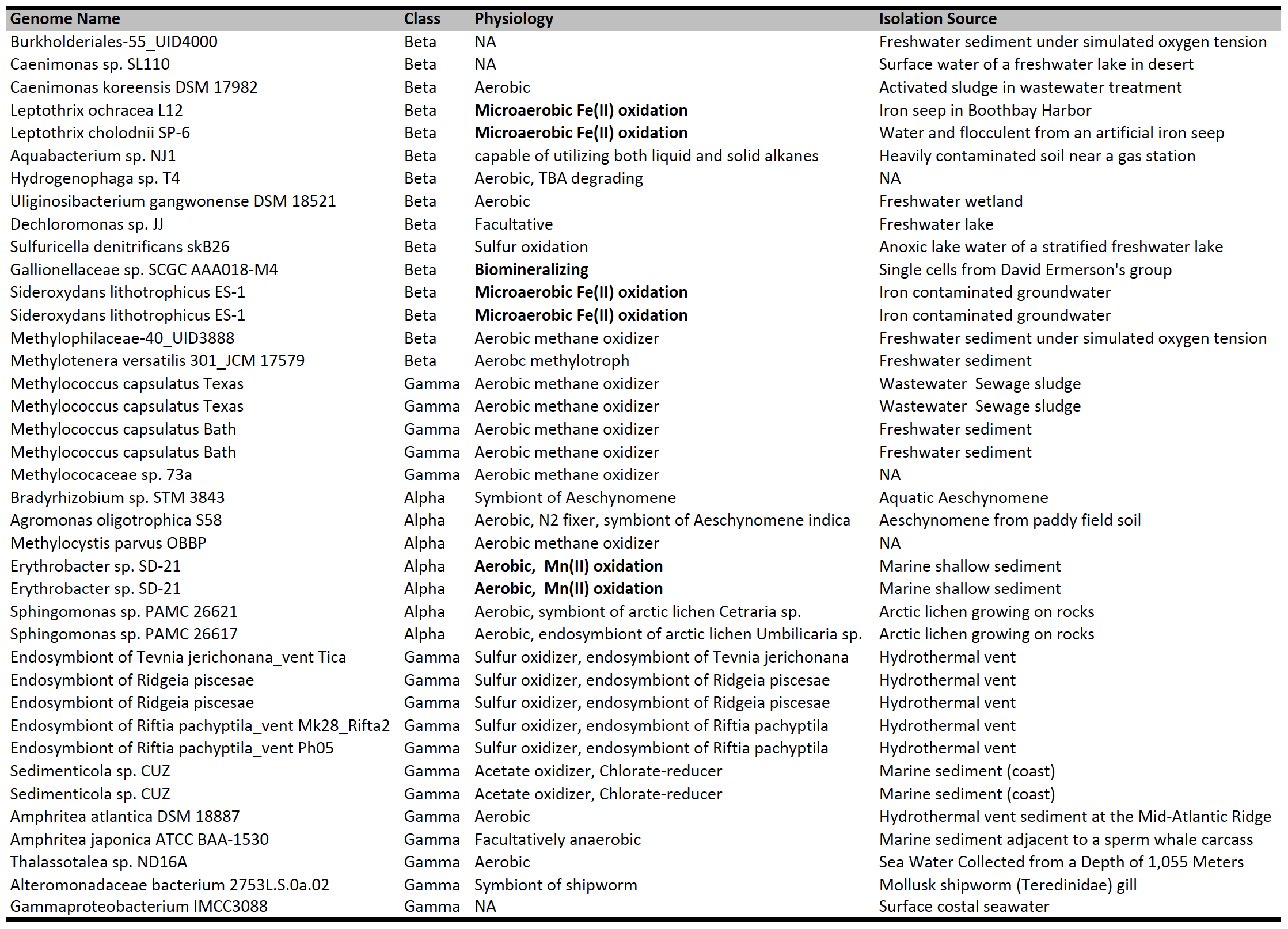


*^a^*Genomes are ordered based on their porin phylogenetic positions in the tree in Figure S1.

*^b^*NA: not available.
