# Supplementary material for: Comparative Genomic Analysis of Neutrophilic Iron(II) Oxidizer Genomes for Candidate Genes in Extracellular Electron Transfer
Source: Front Microbiol. 2017 Aug 21;8:1584. doi: 10.3389/fmicb.2017.01584 (PMC5566968; doi:10.3389/fmicb.2017.01584)
Supplement: Supplementary file 4 [file Table4.DOCX]

**Supplementary Table 4. The four component genes within the PCC3 cluster**


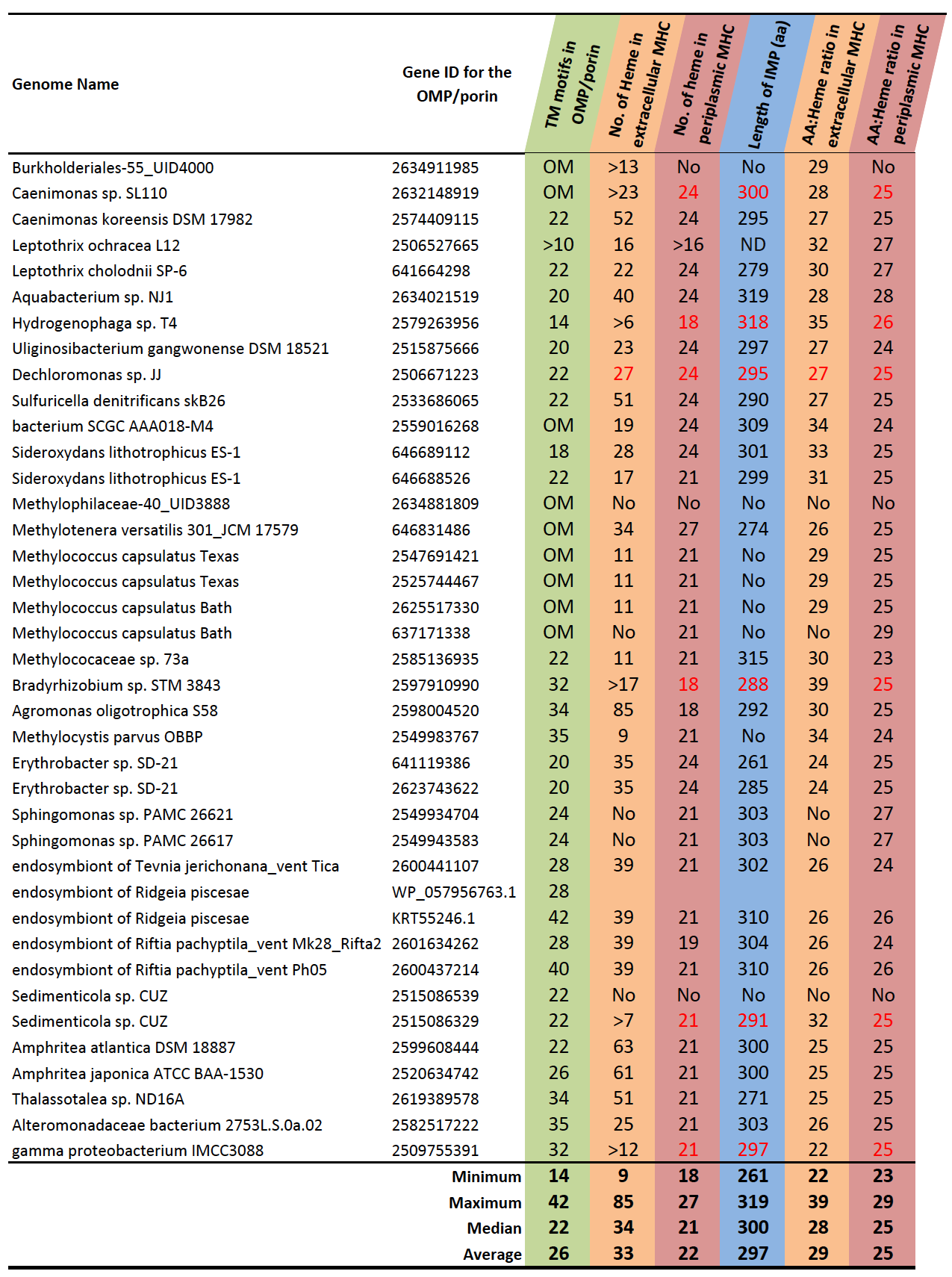


Genes are ordered based on positions in Figure S1. IMG gene ID or GenBank accession numbers for the OMP/porin are listed.

**TM motifs in OMP/porin:** The number of transmembrane (TM) motifs in the OMP/porin. If this cell is “OM”, it means that this protein is predicted be an outer membrane protein, but is unlikely to form a porin due to the very few TM motifs.

**No. of Heme in extracellular MHC** and **No. of Heme in periplasmic MHC:** The number of heme-binding sites in the extracellular and periplasmic MHCs, respectively. “No” means that no homologous protein was found in the genome. Numbers in red mean that a homolog was found, but is at the edge of another contig, which could have been joined to this contig.

**Length of IMP**: The length of the IMP. If this cell is “No”, it means that no homolog was found in the genome.

**AA: Heme ratio in extracellular MHC** and **AA: Heme ratio in periplasmic MHC:** The amino acid-to-heme ratio for the extracellular and periplasmic MHCs, respectively, calculated by dividing the protein length by the number of heme-binding sites.
