# Supplementary material for: Comparative Genomic Analysis of Neutrophilic Iron(II) Oxidizer Genomes for Candidate Genes in Extracellular Electron Transfer
Source: Front Microbiol. 2017 Aug 21;8:1584. doi: 10.3389/fmicb.2017.01584 (PMC5566968; doi:10.3389/fmicb.2017.01584)
Supplement: Supplementary file 6 [file Table6.DOCX]

**Supplementary Table 6. Gene cluster encoding a multiheme cytochrome *c*, an inner membrane protein with two conserved HX­_13_H motifs and a redox protein, but without a porin-coding gene in the same cluster**

| **Genome ID** | **Genome Name** | **Multiheme cytochrome *c*** | | | | **Inner membrane protein** | | | | **Other redox protein** | |
| --- | --- | --- | --- | --- | --- | --- | --- | --- | --- | --- | --- |
|  |  | Gene ID | Length (aa) | Heme-binding sites | Length (aa) to heme ratio | Gene ID | Length (aa) | Predicted trans-membrane helix | Two conserved HX_13_H motifs | Gene ID | Length (aa) |
| 2529292555 | *Bradyrhizobium japonicum* 22 | 2529391855 | 258 | 7 | 37 | 2529391856 | 235 | 5 | Yes | Absent |  |
| 2529292555 | *Bradyrhizobium japonicum* 22 | 2529391974 | 256 | 7 | 37 | 2529391975 | 235 | 5 | Yes | 2529391976 | 608 |
| 2524614546 | *Bradyrhizobium japonicum* in8p8 | 2524882968 | 258 | 7 | 37 | 2524882967 | 234 | 5 | Yes | 2524882966 | 687 |
| 2524614546 | *Bradyrhizobium japonicum* in8p8 | 2524883054 | 226 | 8 | 28 | 2524883055 | 195 | 4 | Yes | Absent |  |
| 2524614545 | *Bradyrhizobium japonicum* is5 | 2524875418 | 226 | 8 | 28 | 2524875417 | 195 | 4 | Yes | Absent |  |
| 2524614545 | *Bradyrhizobium japonicum* is5 | 2524876994 | 258 | 7 | 37 | 2524876995 | 234 | 5 | Yes | 2524876996 | 687 |
| 642555153 | *Rhodopseudomonas palustris* TIE-1 | 642710993 | 258 | 7 | 37 | 642710992 | 231 | 5 | Yes | 642710991 | 702 |
| 2528768215 | *Dechloromonas agitata* is5 | 2529237647 | 249 | 8 | 31 | 2529237648 | 192 | 5 | Yes | 2529237649 | 425 |
| 648028028 | *Gallionella capsiferriformans* ES-2 | 648148335 | 247 | 8 | 31 | 648148334 | 192 | 5 | Yes | 648148333 | 424 |
| 648028028 | *Gallionella capsiferriformans* ES-2 | 648149105 | 544 | 9 | 60 | 648149104 | 308 | 5 | Yes | 648149103 | 796 |
| 2264867016 | *Gallionella* sp. SCGC AAA018-N21 | 2264885405 | 555 | 9 | 62 | 2264885406 | 310 | 5 | Yes | 2264885407 | 805 |
| 2565956535 | *Gallionellaceae* sp. in NDFO enrichment | 2566081409 | 556 | 9 | 62 | 2566081408 | 308 | 5 | Yes | 2566081407 | 805 |
| 2565956535 | *Gallionellaceae* sp. in NDFO enrichment | 2566083217 | 252 | 8 | 32 | 2566083218 | 192 | 5 | Yes | Absent |  |
| 641522635 | *Leptothrix cholodnii* SP-6 | 641663890 | 288 | 10 | 29 | 641663891 | 192 | 5 | Yes | 641663892 | 412 |
| 638341060 | *Chlorobium ferrooxidans* DSM 13031 | 639205725 | 471 | 18 | 26 | 639205724 | 192 | 4 | Yes | Absent |  |
