# Supplementary material for: Comparative Genomic Analysis of Neutrophilic Iron(II) Oxidizer Genomes for Candidate Genes in Extracellular Electron Transfer
Source: Front Microbiol. 2017 Aug 21;8:1584. doi: 10.3389/fmicb.2017.01584 (PMC5566968; doi:10.3389/fmicb.2017.01584)
Supplement: Supplementary file 7 [file Table7.DOCX]

**Supplementary Table 7. Occurrence of PcoAB gene cluster in *Bradyrhizobium japonicum* genomes available in the IMG database**

| **Genome** | **PcoAB** |
| --- | --- |
| *Bradyrhizobium japonicum* 22 | 1 |
| *Bradyrhizobium japonicum* in8p8 | 1 |
| *Bradyrhizobium japonicum* is5 | 1 |
| *Bradyrhizobium japonicum* CCBAU 15354 | 0 |
| *Bradyrhizobium japonicum* CCBAU 15517 | 0 |
| *Bradyrhizobium japonicum* CCBAU 15618 | 0 |
| *Bradyrhizobium japonicum* CCBAU 25435 | 0 |
| *Bradyrhizobium japonicum* CCBAU 83623 | 0 |
| *Bradyrhizobium japonicum* E109 | 0 |
| *Bradyrhizobium japonicum* FN1 | 0 |
| *Bradyrhizobium japonicum* Is-1 | 0 |
| *Bradyrhizobium japonicum* Is-34 | 0 |
| *Bradyrhizobium japonicum* NK6 | 0 |
| *Bradyrhizobium japonicum* SEMIA 5079 | 0 |
| *Bradyrhizobium japonicum* SEMIA 5080 | 0 |
| *Bradyrhizobium japonicum* USDA 110 | 0 |
| *Bradyrhizobium japonicum* USDA 122 | 0 |
| *Bradyrhizobium japonicum* USDA 123 | 0 |
| *Bradyrhizobium japonicum* USDA 124 | 0 |
| *Bradyrhizobium japonicum* USDA 135 | 0 |
| *Bradyrhizobium japonicum* USDA 38 | 0 |
| *Bradyrhizobium japonicum* USDA 4 | 0 |
| *Bradyrhizobium japonicum* USDA 6 | 0 |
