# Supplementary material for: Comparative Genomic Analysis of Neutrophilic Iron(II) Oxidizer Genomes for Candidate Genes in Extracellular Electron Transfer
Source: Front Microbiol. 2017 Aug 21;8:1584. doi: 10.3389/fmicb.2017.01584 (PMC5566968; doi:10.3389/fmicb.2017.01584)
Supplement: Supplementary file 8 [file Image1.PDF]

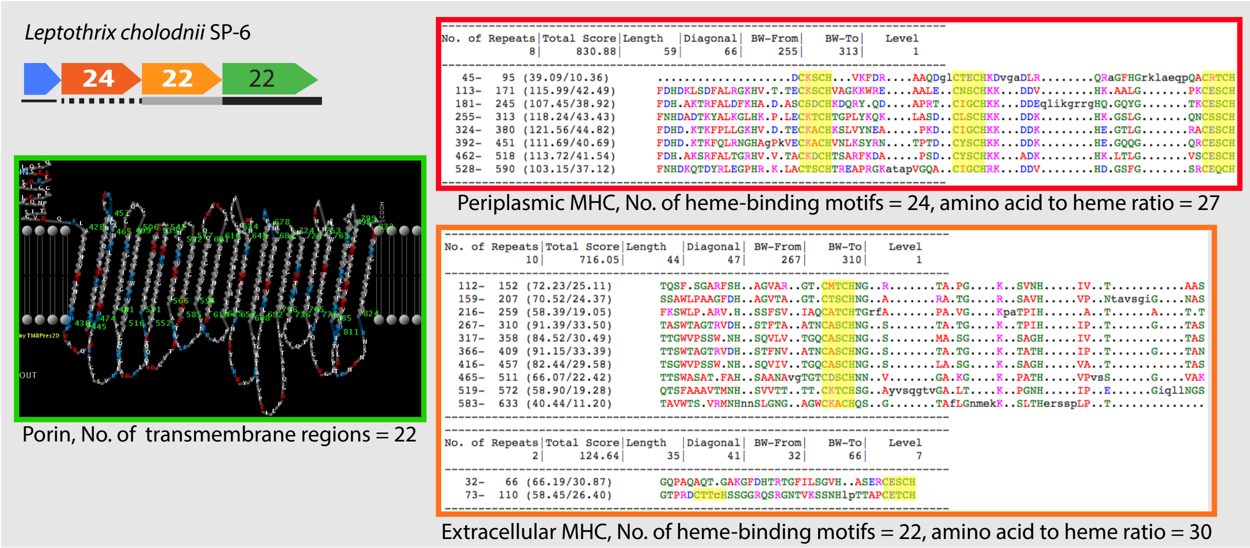

(A)

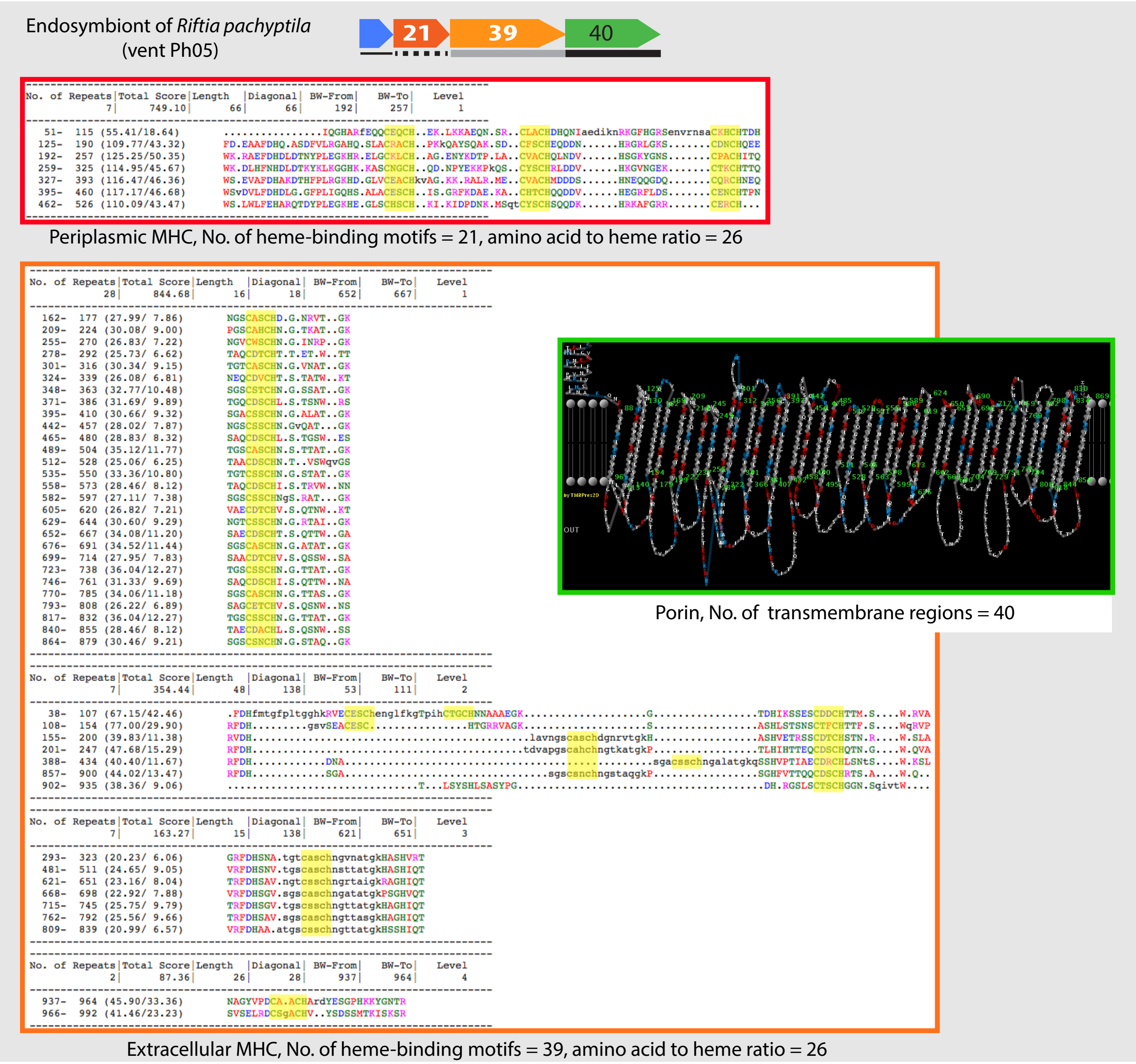

(B)

Supplementary Figure 1. Sequence repeats within the extracellular and periplasmic MHCs and the predicted 2D-representation of the beta barrel structure of the porin in the PCC3 gene cluster of Leptothrix cholodnii SP-6 (**A**) and an endosymbiont of Riftia pachyptila from a hydrothermal vent (**B**), respectively. Sequence repeats were predicted by RADAR (Rapid Automatic Detection and Alignment of Repeats), and the 2D-representaiton of porin was predicted by PRED-TMBB. Heme-binding sites are highlighted in yellow.
